# Integrated Bioinformatics Methods Were Employed to Investigate Potential Molecular Links Between Obstructive Sleep Apnea and Sarcoidosis

**DOI:** 10.1155/mi/7909167

**Published:** 2026-06-18

**Authors:** Yi Li, Chengdian Lan, Haiyan Lei, Zhi Lyu, Danxia Lin, Jiali Shen, Hongzhan Jiang, Yulin Wang, Congying Lu, Yao Liu

**Affiliations:** ^1^ Department of General Practice, Zhongshan Hospital (Xiamen), Fudan University, Xiamen, China, fudan.edu.cn; ^2^ Department of Senior Cadres Ward, Zhongshan Hospital of Xiamen University, School of Medicine, Xiamen University, Xiamen, China, xmu.edu.cn; ^3^ The School of Clinical Medicine, Fujian Medical University, Fuzhou, China, fjmu.edu.cn; ^4^ Department of Hematology, Zhongshan Hospital (Xiamen), Fudan University, Xiamen, China, fudan.edu.cn; ^5^ Nursing Department, The First Affiliated Hospital of Xiamen University, School of Medicine, Xiamen University, Xiamen, China, xmu.edu.cn; ^6^ School of Nursing, Beijing University of Chinese Medicine, Beijing, China, bucm.edu.cn; ^7^ Department of Pulmonary Medicine, Zhongshan Hospital (Xiamen), Fudan University, Xiamen, China, fudan.edu.cn; ^8^ Department of General Practice, Zhongshan Hospital, Fudan University, Shanghai, China, fudan.edu.cn

**Keywords:** computational biology, immunity, inflammation, obstructive sleep apnea, sarcoidosis

## Abstract

**Background:**

Previous studies have established a frequent coexistence of obstructive sleep apnea (OSA) and sarcoidosis, yet the molecular mechanisms linking these conditions are not well understood.

**Methods:**

This study employed integrated bioinformatics analysis and Mendelian randomization (MR) to identify potential mechanisms and infer causal relationships between OSA and sarcoidosis. Genetic instruments associated with OSA and sarcoidosis were screened, and bidirectional MR analysis was conducted to explore the causal relationship between the two conditions. Colocalization analysis was conducted to identify genes potentially associated with both OSA and sarcoidosis. Subsequently, differential expression analysis and weighted gene coexpression network analysis (WGCNA) were performed using data from the Gene Expression Omnibus (GEO) to identify differentially expressed genes (DEGs). In addition, eight machine learning algorithms were used to construct a nomogram based on the importance scores of DEGs and to identify hub genes. The results were validated using multiple methods.

**Results:**

MR analysis confirmed a causal relationship between OSA and sarcoidosis (*p* = 0.0018 OR = 1.39 [95% CI 1.18–1.59]). We identified 35 genes associated with both OSA and sarcoidosis, among which 6 were found to be differentially expressed. KRT72, DEFA4, RNASE3, and LTF were identified as signature genes. Based on these genes, nomograms were constructed to quantitatively predict the risk of comorbid OSA and sarcoidosis.

**Conclusion:**

This study indicates a causal relationship between OSA and sarcoidosis. The potential mechanisms underlying this comorbidity involve immune dysregulation, inflammatory responses, and altered DNA repair and methylation. Additional contributing factors include dysregulated apoptosis, immune microenvironment remodeling, autophagy, and neural activity.

## 1. Introduction

Sarcoidosis is a multisystem granulomatous disorder of unknown etiology, predominantly characterized by lymphadenopathy and noncaseating granuloma formation. At the same time, sarcoidosis exhibits significant geographic variation and interpatient heterogeneity [1]. Furthermore, its typically insidious onset and occasional aggressive clinical course present substantial diagnostic challenges for clinicians and complicate research efforts [2].

Obstructive sleep apnea (OSA) is a global disorder characterized by recurrent episodes of partial or complete upper airway collapse during sleep, leading to disrupted ventilation and oxygen desaturation. Notably, several studies have reported a high prevalence of OSA among patients with sarcoidosis [3, 4]. In a small study involving 84 patients with pathologically confirmed sarcoidosis, 60 were found to have OSA [5]. Another study involving 71 patients with sarcoidosis and 71 matched healthy controls found that patients with sarcoidosis had a 2.5‐fold increased risk of mild OSA compared to controls [6]. However, these studies involved small sample sizes and treated OSA as a complication of sarcoidosis. At the same time, establishing a causal relationship between OSA and sarcoidosis is challenging due to potential confounding bias and reverse causality inherent in observational studies [7].

Currently, the pathogenic mechanism underlying the association between OSA and sarcoidosis remains unclear. Some studies suggest that the development of OSA in patients with sarcoidosis may result from altered airway conditions and reduced lung volumes secondary to pulmonary damage [8, 9]. However, one study found no correlation between lung function and polysomnographic findings in OSA patients with sarcoidosis [10]. The relationship between OSA and sarcoidosis, therefore, remains unclear. Furthermore, current research has not yet identified the molecular mechanisms linking these two conditions. Further research to elucidate the molecular and physiological connections between these factors is therefore warranted [11].

**Figure 1:**
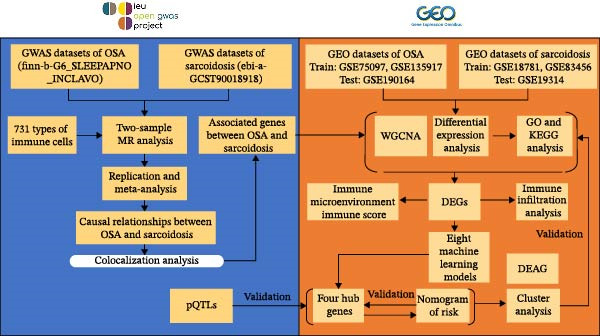
Flowchart of this study design. DEAG, DEAG; DEGs, differentially expressed genes; GEO, Gene Expression Omnibus; GO, Gene Ontology; GWAS, genome‐wide association study; IS, ischemic stroke; KEGG, Kyoto Encyclopedia of Genes and Genomes; MR, Mendelian randomization; OSA, obstructive sleep apnea; pQTL, protein quantitative trait loci; WGCNA, weighted gene coexpression network analysis.

Mendelian randomization (MR) leverages natural genetic variation as instrumental variables (IVs) to infer causal relationships between exposures and outcomes, effectively circumventing reverse causation and confounding biases inherent in conventional observational studies [7]. Colocalization analysis was employed to identify potential genes by examining whether the two traits share common genetic loci, and genes associated with both traits were further screened using data from the Gene Expression Omnibus (GEO) database. This approach not only strengthens the credibility of the research but also serves as a valuable resource for identifying gene‐associated pathways, advancing our understanding of gene‐mediated pathophysiology, and enabling risk prediction for related diseases. Therefore, this study first established the causal relationship between OSA and sarcoidosis using MR and identified genes associated with both conditions through MR and colocalization analyses. Human samples were then used to validate the MR findings and identify key biomarkers for further exploration of the mechanisms underlying OSA in combination with sarcoidosis. Figure 1 presents the workflow of the current study.

## 2. Methods

### 2.1. Data Source

This study utilized previously collected and publicly available datasets, including genome‐wide association study (GWAS) summary statistics and GEO expression data. All original studies received approval from the respective institutional ethics committees, and informed consent was obtained from all participants. In our MR study, GWAS summary data for OSA were obtained from the FinnGen cohort, comprising 16,761 OSA patients and 201,194 controls [12, 13]. The diagnosis of OSA was based on the International Classification of Diseases, 9th and 10th Revisions (ICD‐9:3472A; ICD‐10: G47.3), determined through documentation of subjective symptoms, clinical assessments, and objective measures, including the apnea–hypopnea index (AHI) and respiratory event index. The GWAS summary data for sarcoidosis were derived from a large‐scale GWAS encompassing ~707,000 individuals across multiple biobanks (GWAS ID: ebi‐a‐GCST90018918). These publicly available data were accessed through the IEU OpenGWAS Project (https://gwas.mrcieu.ac.uk). Additionally, immune cell‐related GWAS data were obtained from the GWAS Catalog (https://www.ebi.ac.uk/gwas/downloads/summary-statistics), covering summary statistics with IDs ranging from GCST0001391 to GCST0002121. These datasets encompass 731 immune phenotypes, including absolute cell counts (AC; 118 traits), median fluorescence intensity (MFI) of surface antigen expression (389 traits), morphological parameters (MP; 32 traits), and relative cell counts (RC; 192 traits). The MFI, AC, and RC categories include phenotypes related to B cells, circulating dendritic cells (CDCs), mature T cells, monocytes, bone marrow‐derived cells, TBNK (T cells, B cells, and natural killer cells), and regulatory T cells (Tregs), while MP traits encompass features related to CDCs and TBNKs. The GEO datasets for OSA were obtained from GSE135917 and GSE75097. The GSE135917 dataset includes transcriptomic data from normal controls, OSA patients without continuous positive airway pressure (CPAP) treatment, and OSA patients following CPAP treatment. Specifically, it comprises 8 normal control samples, 33 OSA patient samples without CPAP treatment, and 25 OSA patient samples with CPAP treatment. For the purposes of this study, only the normal control samples and OSA samples without CPAP treatment were selected for downstream analysis. The GSE75097 dataset focuses on sleep apnea syndrome and includes samples categorized by AHI severity: primary snoring (AHI < 5), moderate to severe sleep apnea (15 < AHI ≤ 50), very severe sleep apnea (AHI > 50), and patients with very severe sleep apnea undergoing long‐term CPAP therapy (defined as regular use > 4 h/night for > 1 year). In this study, only the first three categories were included for analysis, while samples from patients under long‐term CPAP treatment were excluded. The OSA validation dataset, GSE190164, included 8 patients diagnosed with OSA using polysomnography (PSG) and 8 healthy control subjects. The GEO datasets GSE18781, GSE83456, and GSE19314 were used for sarcoidosis analysis. GSE18781 includes 12 sarcoidosis samples and 25 normal control samples, while GSE83456 comprises 49 sarcoidosis samples and 86 normal controls. GSE19314 contains 38 sarcoidosis samples and 20 control samples and was designated as the validation dataset. The GEO datasets GSE75097 and GSE135917 were used as test sets for OSA, with GSE190164 serving as the validation set. For sarcoidosis, GSE83456 and GSE19314 were used as test sets, while GSE19314 also served as the validation set. The “sva” and “limma” packages in R were used to remove batch effects and merge the two OSA test sets. The same approach was applied to merge the two test sets of sarcoidosis. The flowchart is shown in Figure 1.

### 2.2. Selection of Genetic IVs

Initially, we selected GWAS data relevant to exposure‐related factors and identified genome‐wide significant genetic loci (*p*  < 5 × 10^−8^). Subsequently, linkage disequilibrium (LD) clumping was performed using thresholds of *r* ^2^ < 0.001 and a window size of 10,000 kilobases to ensure independence among selected single nucleotide polymorphisms (SNPs), thereby minimizing potential confounding due to LD.

Genetic IVs, serving as proxies for the exposure, were required to satisfy three core assumptions: (a) a significant association between genetic instruments and the exposure; (b) no association between the instruments and potential confounders; and (c) the effect on the outcome occurs exclusively through the exposure. Adherence to these assumptions strengthens the validity of the IVs and ensures robust causal inference.

Summary statistics for eligible SNPs were extracted from both exposure and outcome GWAS datasets. Palindromic SNPs were excluded to maintain allele alignment and directional consistency of effect estimates across exposure and outcomes.

Following this rigorous selection process, the finalized SNPs were used as genetic instruments for MR analyses in this study.

### 2.3. MR Statistical Analysis

The “TwoSampleMR” package was used to select relevant SNPs as IVs for two‐sample bidirectional MR, employing inverse variance weighting (IVW), the MR‐PRESSO method to account for pleiotropy and outliers, MR‐Egger regression, and the weighted median method. IVW was the main method used. Additionally, MR‐PRESSO and MR‐Egger were used to assess horizontal pleiotropy. A *p*‐value of < 0.05 was considered indicative of the presence of horizontal pleiotropy. Cochran’s *Q* test for the IVW method was used to detect heterogeneity. Precisely, if the *p*‐value from the Cochran’s *Q* test was greater than 0.05, no heterogeneity was observed. Finally, the leave‐one‐out method was employed to assess the impact of individual SNPs on the results of the MR analysis.

### 2.4. Repeat MR Analysis and Meta‐Analysis

We replicated the MR analysis in an independent, predominantly European cohort comprising 13,818 OSA patients and 463,035 controls from the UK Biobank [14]. Since an insufficient number of SNPs were identified at the genome‐wide significance threshold (*p*  < 5 × 10^−8^), we relaxed the threshold to *p*  < 5 × 10^−6^ to extract additional IVs. A meta‐analysis of the MR results from the two cohorts was subsequently performed using the “meta” package in R. A two‐sided *p*‐value < 0.05 was considered statistically significant.

### 2.5. OSA‐Sarcoidosis Colocalization Analysis

We performed colocalization analysis using the “coloc” package to identify shared genetic variants between OSA and sarcoidosis. The results indicated that most of the shared loci between the two traits may be driven by either common or distinct SNPs. A locus was considered colocalized if the posterior probability for hypothesis 4 (PPH4) exceeded 0.7. Genes located within these genomic regions were identified as signature genes shared by OSA and sarcoidosis.

### 2.6. Differential Expression Analysis

To identify differentially expressed genes (DEGs) in OSA and sarcoidosis, we utilized the “limma” package in R [15], which performs differential expression analysis of OSA and sarcoidosis samples relative to normal controls. DEGs were defined as genes with |log_2_FC| > 0.5 and *p*  < 0.05.

### 2.7. Weighted Gene Coexpression Network Analysis (WGCNA) Analysis

WGCNA offers a systematic approach to explore gene–clinical trait relationships by organizing coexpressed genes into discrete modules, which often correspond to distinct biological pathways. This method was employed to assess associations between gene networks and clinical traits—specifically, OSA and sarcoidosis.

The analytical pipeline began with the construction of a sample dendrogram and hierarchical clustering via the flashClust function in R, including outlier detection and removal to ensure data quality. The optimal soft‐thresholding power (selected from an empirical range of 1–30) was determined using the WGCNA package to achieve a scale‐free network topology.

An adjacency matrix was generated based on pairwise gene coexpression, which was then transformed into a topological overlap matrix (TOM) to more accurately reflect gene‐gene connectivity while reducing noise from spurious correlations. Modules of coexpressed genes were identified using dynamic tree‐cutting algorithms (with a minimum module size of 30 genes). Finally, module–trait relationships were systematically evaluated to identify modules significantly associated with clinical phenotypes of interest. DEGs were defined as genes with module eigengene connectivity (kME) absolute values ≥0.8.

### 2.8. Gene Ontology (GO) and Kyoto Encyclopedia of Genes and Genomes (KEGG) Pathway Analysis

Functional enrichment analysis of the DEGs was performed using the DAVID online tool (https://david.ncifcrf.gov/) [16]. GO analysis was conducted to annotate genes in three categories: biological process (BP), cellular component (CC), and molecular function (MF), thereby revealing their biological characteristics. Additionally, KEGG pathway analysis was used to identify signaling and metabolic pathways associated with the DEGs.

### 2.9. Dentification and Validation of Hub Genes Using Machine Learning and Construction of Nomograms

We constructed eight prediction models using different machine learning algorithms: least absolute shrinkage and selection operator (LASSO), neural network (NNET), extreme gradient boosting (XGB), support vector machine (SVM), random forest (RF), K‐nearest neighbors (KNN), decision tree (DT), and generalized linear model (GLM). We identified the most appropriate prediction model for each disease based on receiver operating characteristic (ROC) curves and used Venn diagrams to determine the intersecting genes across the optimal models, thereby identifying hub genes. Nomograms were then constructed for the hub gene in both OSA and sarcoidosis. To validate the accuracy of the hub gene‐based prediction models, we also identified the most appropriate prediction models for OSA and sarcoidosis using the validation datasets. ROC curves were then used to assess their performance and applicability in the validation sets.

### 2.10. pQTL Colocalization Analysis

Given that we previously identified shared genetic variants between OSA and sarcoidosis at the genomic level, we further validated the causal associations of hub genes with OSA and sarcoidosis at the protein level using cis‐pQTL data from a recent study [17]. Colocalization analysis was performed for OSA and sarcoidosis using the “coloc” package in R. Colocalization analysis is based on the following five hypotheses: H0: There is no significant association between phenotype 1 and phenotype 2 among all SNP sites in the genomic region. H1/H2: either phenotype 1 or phenotype 2 shows a significant association at all SNP sites in the genomic region. H3: There is a significant association between phenotype 1 and phenotype 2 at all SNP sites in a genomic region, but it is driven by different causal variant sites. H4: There is a significant association between phenotype 1 and phenotype 2 at all SNP sites in a genomic region, and it is driven by the same causal variant site. We set a posttest probability of colocalization at 70%, indicating that genetic exposure in this region is associated with both OSA and sarcoidosis.

### 2.11. Immune Microenvironment Immune Score

We used the “estimate” package to analyze the immune cell status in OSA and sarcoidosis, evaluate the proportions of immune cells, and calculate immune scores. A higher immune score indicates a greater infiltration of immune cells. Additionally, box plots were generated using the “ggpubr” package to visualize the score differences between the disease and control groups. A *p*‐value < 0.05 was considered statistically significant.

### 2.12. Correlation Between Immune Cells and DEGs

Immune cell infiltration was analyzed using the “CIBERSORT” package, followed by Spearman correlation analysis to assess the relationship between immune cell types and the expression levels of DEGs.

### 2.13. Cluster Analysis of DEGs and Analysis Between Expression Clusters

We performed consensus clustering analysis on both OSA and sarcoidosis samples, identifying up to nine distinct clusters. Box plots were then used to compare the expression differences of DEGs across these clusters. Additionally, the “CIBERSORT” algorithm was used to investigate differences in immune cell infiltration among different clusters in both OSA and sarcoidosis. Furthermore, gene set variation analysis (GSVA) was conducted to compare expression patterns between clusters, based on GO and KEGG GMT files downloaded from the GSEA platform and analyzed using R. In addition, we performed differential expression analysis on the OSA and sarcoidosis clusters to identify disease‐differentially expressed associated genes (DEAG), using |logFC| > 1 and *p*  < 0.05 as the screening criteria. The intersecting DEAGs associated with both OSA and sarcoidosis were identified using a Venn diagram, and the results were visualized with heat maps.

### 2.14. DEAG Cluster Analysis and Intercluster Analysis

We conducted a second clustering analysis of OSA and sarcoidosis samples based on the expression of DEAG using the same clustering method as in the initial analysis. We further examined the expression levels of DEGs within DEAG‐defined clusters and analyzed the immune cell infiltration across different clusters.

### 2.15. Construction of DEGs Clustering Score

Following the method described by Liu et al. [18], we applied the principal component analysis (PCA)‐based scoring approach to construct DEGs scores in OSA and sarcoidosis samples, respectively. The scoring formula, derived from the expression levels of DEGs in each sample, is as follows: DEG score = ∑ (PC1i + PC2i). Furthermore, we performed differential analysis of the DEGs and DEAG cluster scores in both OSA and sarcoidosis samples. The results were visualized using box plots. To visually illustrate the associations between DEGs clusters, DEAG clusters, and DEGs clustering scores, we generated two alluvial plots representing these relationships in OSA and sarcoidosis, respectively.

### 2.16. Statistical Analysis

All analyses were primarily conducted using R software (Version 4.4.1). In the colocalization analysis, chromosomal loci were defined within a range of 75–500 kilobases to maximize the identification of trait‐associated genes. For comparisons between two independent groups, Student’s *t*‐test was applied. The Wilcoxon signed‐rank test was used for paired sample comparisons. For comparisons involving three or more groups, either analysis of variance (ANOVA) or the Kruskal–Wallis rank‐sum test was employed, depending on the distribution and variance of the data. Spearman rank correlation was used to assess the associations between variables. A *p*‐value < 0.05 was considered statistically significant.

## 3. Results

### 3.1. MR Analysis and Meta‐Analysis Results

**Figure 2:**
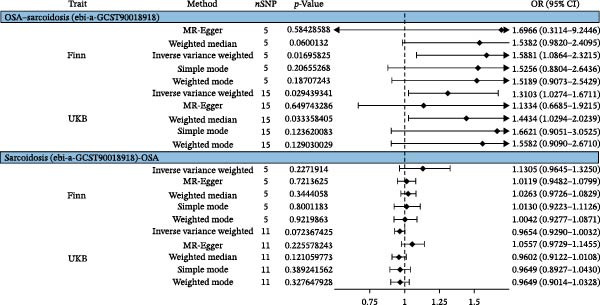
Forest plot of Mendelian randomization (MR) analysis between OSA (obstructive sleep apnea) and sarcoidosis. CI, confidence interval; OR, odds ratio; OSA, obstructive sleep apnea.

We used IVW analysis as the primary method, with MR‐Egger, weighted median, and MR‐PRESSO as supplementary methods. The results of the bidirectional MR analysis are presented in Figure 2 and Table 1. Meanwhile, the *F*‐statistics for the selected SNPs all exceeded 10, and the *p*‐values from both the MR‐Egger and Cochran’s *Q* tests were greater than 0.05. These results confirm that the IVs satisfied the three core MR assumptions. After excluding heterogeneity and horizontal pleiotropy, our analysis confirmed a causal relationship in which OSA, as the exposure, was a risk factor for sarcoidosis (*p* = 0.0018, OR = 1.39, 95% CI: 1.18–1.59). However, no causal association was found in the reverse direction, indicating that sarcoidosis is not a risk factor for OSA (*p* = 0.36 OR = 0.99 95% CI: 0.95–1.02). Moreover, repeated analyses yielded consistent results (Table 2). A meta‐analysis of the two bidirectional MR studies further confirmed that OSA, as the exposure, has a causal relationship with sarcoidosis (*p*  < 0.05) (Table 3). SNP‐related information can be seen in Tables S1–S4.

**Table 1:** MR analysis between OSA and sarcoidosis.

Exposure	Outcome	MR methods	*N* SNPs	OR (95% CI)	*p*‐Value	MR‐Egger Q_*p*val‐value	MR‐Egger_intercept *p*‐value
OSA	Sarcoidosis	IVW	5	1.5881 (1.0864–2.3215)	0.01694	0.9865	0.9424
OSA	Sarcoidosis	Weighted median	5	1.5382 (0.9820–2.4095)	0.06	—	—
OSA	Sarcoidosis	MR‐Egger	5	1.6966 (0.3114–9.2446)	0.5843	—	—
OSA	Sarcoidosis	Simple mode	5	1.5256 (0.8804–2.6436)	0.2066	—	—
OSA	Sarcoidosis	Weight mode	5	1.5189 (0.9073–2.5429)	0.1871	—	—
Sarcoidosis	OSA	IVW	5	1.1305 (0.9645–1.3250)	0.2272	0.9713	0.2939
Sarcoidosis	OSA	Weighted median	5	1.0263 (0.9726–1.0829)	0.3441	—	—
Sarcoidosis	OSA	MR‐Egger	5	1.0119 (0.9482–1.0799)	0.7214	—	—
Sarcoidosis	OSA	Simple mode	5	1.0130 (0.9223–1.1126)	0.8012	—	—
Sarcoidosis	OSA	Weight mode	5	1.0042 (0.9277–1.0871)	0.922	—	—

**Table 2:** Repeat MR analysis between OSA and sarcoidosis.

Exposure	Outcome	MR methods	*N* SNPs	OR (95% CI)	*p*‐Value	MR‐Egger Q_*p*val‐value	MR‐Egger_intercept *p*‐value
OSA	Sarcoidosis	IVW	15	1.3103 (1.0274–1.6711)	0.0294	0.6345	0.5544
OSA	Sarcoidosis	Weighted median	15	1.4434 (1.0294–2.0239)	0.0334	—	—
OSA	Sarcoidosis	MR‐Egger	15	1.1334 (0.6685–1.9215)	0.6497	—	—
OSA	Sarcoidosis	Simple mode	15	1.6621 (0.9051–3.0525)	0.1236	—	—
OSA	Sarcoidosis	Weight mode	15	1.5582 (0.9090–2.6710)	0.129	—	—
Sarcoidosis	OSA	IVW	11	0.9654 (0.9290–1.0032)	0.0724	0.9794	0.0579
Sarcoidosis	OSA	Weighted median	11	0.9602 (0.9122–1.0108)	0.344	—	—
Sarcoidosis	OSA	MR‐Egger	11	1.0557 (0.9729–1.1455)	0.2556	—	—
Sarcoidosis	OSA	Simple mode	11	0.9649 (0.8927–1.0430)	0.3892	—	—
Sarcoidosis	OSA	Weight mode	11	0.9649 (0.9014–1.0328)	0.3277	—	—

**Table 3:** MR analysis between OSA and sarcoidosis following meta‐analysis integration.

Exposure	Outcome	OR (95% CI)	*I* ^2^ value	*p*‐Value
OSA	Sarcoidosis	1.39 (1.18–1.59)	0.00%	0.0018
Sarcoidosis	OSA	0.99 (0.95–1.02)	69.62%	0.36

### 3.2. Colocalization Analysis

Colocalization analysis was performed to identify genomic regions with shared genetic variation that may contribute to the association between the two traits. Genes located within regions of shared genetic variation are considered potential contributors to both OSA and sarcoidosis. Most of the shared loci between the two traits are represented by four common candidate causal SNPs with strong colocalization evidence (PPH4 > 0.95) and 15 additional potential candidate causal SNPs (PPH4 > 0.75), as detailed in Table 4. The genes associated with both OSA and sarcoidosis that were identified in these regions are listed in Table 5.

**Table 4:** Posterior probability test of shared loci between OSA and sarcoidosis.

SNP	PPH4
rs114983649	0.96
rs116182122	0.78
rs116420235	0.91
rs142416325	0.94
rs145058358	0.94
rs113955098	0.85
rs115031841	0.86
rs115359853	0.99
rs117575878	0.85
rs12493320	0.92
rs138235930	0.94
rs144191169	0.97
rs17168971	0.75
rs189327915	0.79
rs60700772	0.91
rs61873510	0.99
rs7093230	0.8
rs72981098	0.75
rs7567133	0.85

**Table 5:** Related genes obtained from shared genetic loci between OSA and sarcoidosis.

Genes
PTH2R, KAZN, REL, MAPK9, RARB, CACNB2, NRXN1, SPOCK1, CPQ, CMSS1, PDE10A, MSR1, BMP6, PAX2, FRMD4A, PTPRS, SLC9A2, MEOX2, KRT72, DEFA4, RNASE3, CEACAM8, NT5E, LTF, CAPZBP1, CPNE8, RP11, CTC, NCOA4P1, LINC02315, MIR9, RP5, CASC15, RP1, LINC01016

### 3.3. Results of DEGs Identification

**Figure 4:**
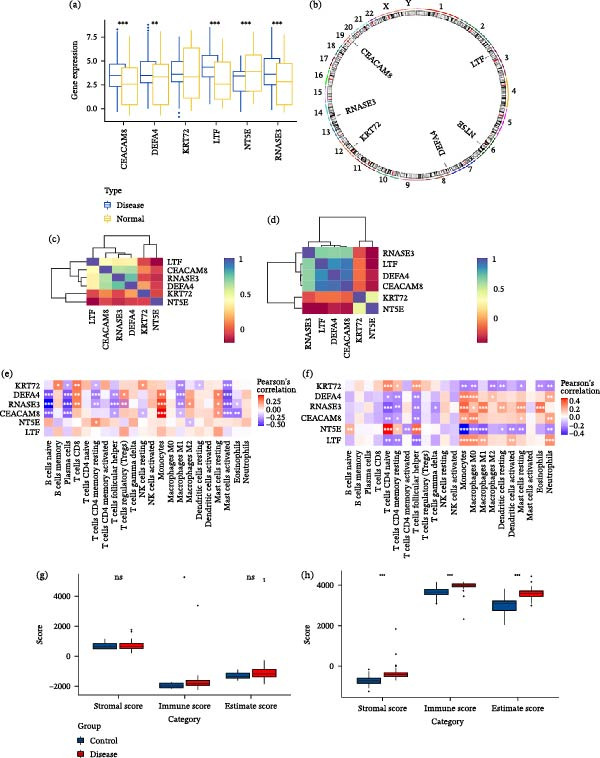
Analysis of DEGs (*n* = 6). (a) Box plot of expression difference analysis of DEGs between the control group and OSA combine sarcoidosis (Wilcoxon rank‐sum test); (b) circle plot of chromosome location of DEGs; (c, d) DEGs correlation heat map (c: OSA, d: sarcoidosis); (e) heat map of correlation analysis between DEGs and immune cells in OSA sample; (f) heat map of correlation analysis between DEGs and immune cells in sarcoidosis sample; (g) box plot of immune score in OSA and control group; (h) box plot of immune score in sarcoidosis and control group. NK indicates natural killer; and Treg, T regulatory.  ^∗^ *p* < 0.05,  ^∗∗^ *p* < 0.01, and  ^∗∗∗^ *p* < 0.001.

We combined the two OSA/sarcoidosis datasets for differential expression analysis and eliminated batch effects (Figure 3a,b). In the differential expression analysis, we identified 16,082 OSA‐related DEGs and 97 sarcoidosis‐related DEGs, with 88 overlapping DEGs obtained through Venn diagram intersection (Figure 3c,d,i). In the WGCNA analysis, we first determined the optimal soft‐thresholding power for the combined dataset of OSA and sarcoidosis and then constructed heat maps illustrating the correlations between module eigengenes and clinical traits (Figure 3e,f). In OSA, the MEgreen and MEblack modules were identified as modules of interest, while in sarcoidosis, the MEblack module was selected (Figure 3g,h). From these key modules, 2194 and 980 genes were identified as module‐related DEGs in OSA and sarcoidosis, respectively. A total of 250 overlapping DEGs were then identified through Venn diagram intersection (Figure 3j). Finally, six DEGs were obtained by overlapping the 250 module‐related DEGs with the previously identified 88 DEGs (Figure 3k). Subsequently, these DEGs were evaluated for overlap with the genes associated with both OSA and sarcoidosis identified in the previous step to determine the final DEGs. In our analysis, a total of six DEGs were identified from the signature genes. Among them, CEACAM8, DEFA4, KRT72, LTF, and RNASE3 were upregulated in both OSA and sarcoidosis, whereas NT5E was downregulated in both conditions (Figure 4a). We visualized the DEGs on the chromosomes, and the specific locations of DEGs on the chromosomes can be seen in Figure 4b. We also conducted a correlation analysis of DEGs, as presented in Figure 4c,d, which revealed that the majority of relationships among DEGs were positively correlated.

Figure 3Identification of differentially expressed genes (DEGs) in OSA (obstructive sleep apnea) and sarcoidosis. (a) PCA plots of the combined OSA datasets before and after batch effects removal. Data 1: GSE135917; data 2: GSE75097; (b) PCA plots of the combined sarcoidosis datasets before and after batch effects removal. Data1: GSE83456; data 2: GSE18781; (c) volcano plot of all DEGs in OSA; (d) volcano plot of all DEGs in sarcoidosis; (e, f) determination of the optimal soft threshold and network connectivity under different soft thresholds; (g, h) correlation heat map of gene modules and clinical traits; (i) Venn diagram showing the intersected numbers of DEGs in OSA and sarcoidosis in differential expression analysis. (j) Venn diagram showing the intersected numbers of DEGs (|kME|≥ 0.8) in OSA and sarcoidosis in WGCNA analysis. (k) Venn diagram showing the total and intersected numbers of DEGs in OSA and sarcoidosis in WGCNA and differential expression analysis. (l) The functional enrichment analysis of common DEGs between OSA and sarcoidosis about differentially expressed analysis; (m) the functional enrichment analysis of common DEGs between OSA and sarcoidosis about WGCNA analysis.

**Figure:**
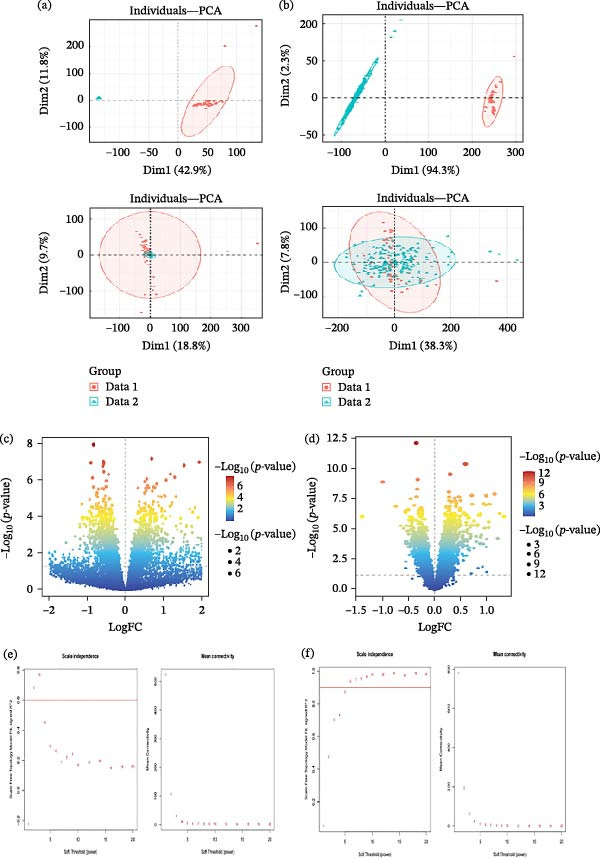

**Figure:**
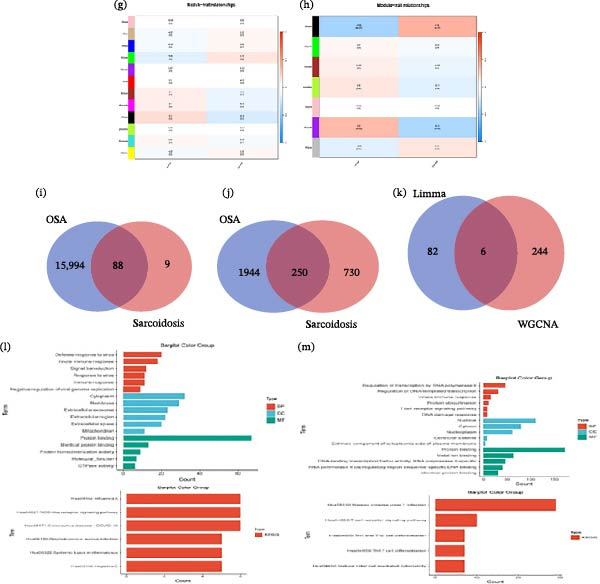

### 3.4. Results of GO and KEGG Pathway Analysis

GO and KEGG pathway enrichment analyses were performed on the DEGs identified through both WGCNA (Figure 4m) and differential expression analyses (Figure 4l). GO enrichment analysis of the DEGs from the differential expression analysis revealed significant enrichment in “innate immune response” and “defense response to virus” within the BP category. In the CC category, DEGs were primarily associated with the cytoplasm, while in the MF category, they were mainly involved in protein binding. Moreover, KEGG pathway analysis indicated that the DEGs were significantly enriched in the NOD‐like receptor signaling pathway. On the other hand, GO enrichment analysis of the DEGs identified through WGCNA revealed that they were primarily associated with “regulation of transcription by RNA polymerase II” and “innate immune response” in the BP category, “cytosol” in the CC category, and “protein binding” in the MF category. KEGG pathway analysis further indicated enrichment in immune‐related pathways, including “natural killer cell‐mediated cytotoxicity,” “Th1 and Th2 cell differentiation,” and the “T cell receptor signaling pathway.”

### 3.5. Mechanism Analysis of the Feature Genes

**Figure 7:**
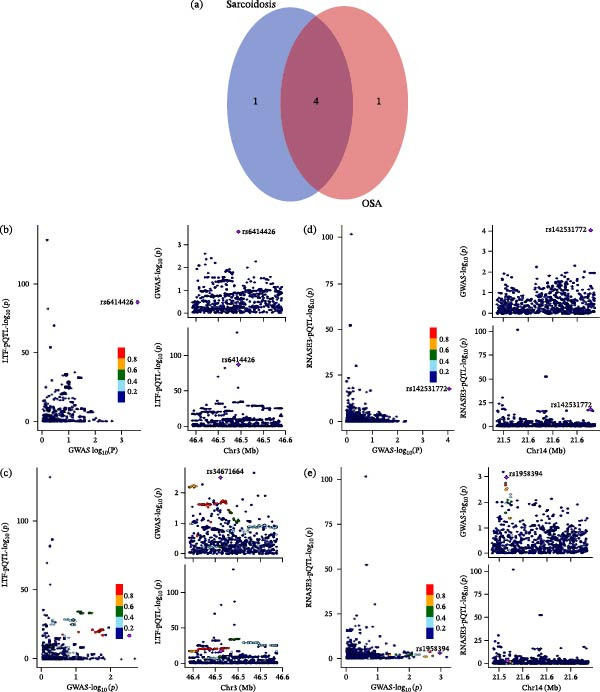
Identification of hub genes and colocalization validation of pQTL. (a) The final candidate biomarkers were overlapped by Venn diagram in OSA and sarcoidosis. Results of LTF (b, c) and RNASE3 (d, e) validation colocalization analysis. Obstructive sleep apnea: (b, d); sarcoidosis: (c, e).

We constructed eight distinct machine learning models using OSA and sarcoidosis sample data, respectively. In the OSA sample, the KNN model exhibited the lowest residual error and the highest area under the ROC curve (Figure 5a,d) and was therefore selected for subsequent analysis. In the sarcoidosis samples, the LASSO and GLM models exhibited the largest areas under the ROC curve and had similar residual values (Figure 6a,d). Therefore, these two models were selected for further analysis. Subsequently, the importance scores of the signature genes were determined in OSA samples and sarcoidosis samples. Furthermore, the characteristic genes identified in OSA and sarcoidosis were intersected using a Venn diagram, resulting in four final signature genes: RNASE3, KRT72, LTF, and DEFA4 (Figure 7a). Detailed information is provided in Tables 6–8. To evaluate the diagnostic performance of the hub genes for OSA and sarcoidosis, nomograms were constructed based on the four genes, respectively. The KNN model was subsequently validated using the OSA validation dataset, while the LASSO and GLM models were validated using the sarcoidosis validation dataset. The results showed that the AUC for the KNN model in OSA samples was 0.906 (Figure 5g), while the LASSO and GLM models achieved AUCs of 0.848 and 0.833, respectively, in sarcoidosis samples (Figure 6g). These findings indicate that diagnostic models based on the selected hub genes demonstrate excellent accuracy in identifying both OSA and sarcoidosis.

Figure 5Machine learning analysis and nomogram construction and validation based on differentially associated genes (DEGs) in the OSA dataset. (a) The OSA dataset’s cumulative residual distribution; (b) the OSA dataset’s inverse cumulative distribution of residuals; (c) important features in the OSA dataset for the DT, GLM, KNN, LASSO, NEET, RF, SVM, and XGB machine models; (d) analysis of the OSA dataset’s receiver operator characteristic (ROC); (e) nomogram of the feature genes; (f) calibration curve; (g) ROC of the validation Gene Expression Omnibus (GEO) data set.

**Figure:**
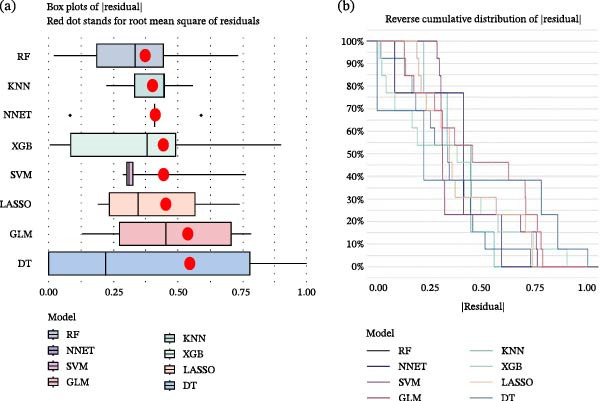

**Figure:**
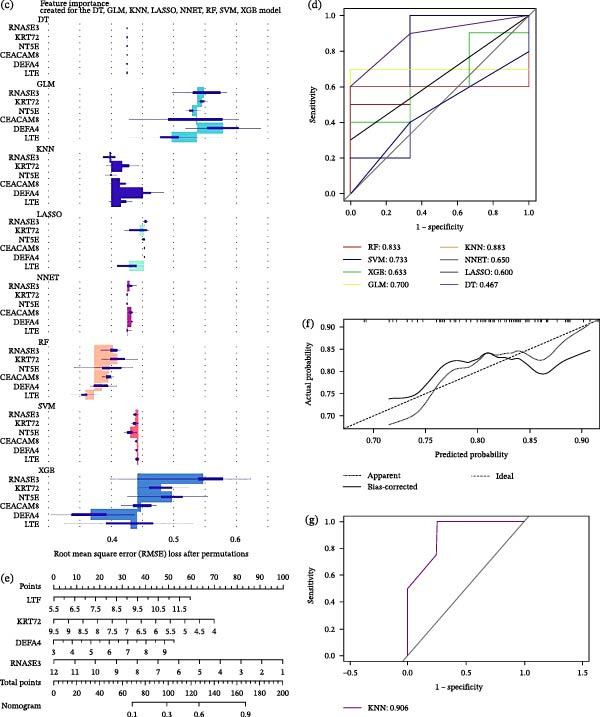

Figure 6Machine learning analysis and nomogram construction and validation based on differentially associated genes (DEGs) in the sarcoidosis dataset. (a) The sarcoidosis dataset’s cumulative residual distribution; (b) the sarcoidosis dataset’s inverse cumulative distribution of residuals; (c) important features in the sarcoidosis dataset for the DT, GLM, KNN, LASSO, NEET, RF, SVM, and XGB machine models; (d) analysis of the sarcoidosis dataset’s receiver operator characteristic (ROC); (e) nomogram of the feature genes; (f) calibration curve; (g) ROC of the validation Gene Expression Omnibus (GEO) data set.

**Figure:**
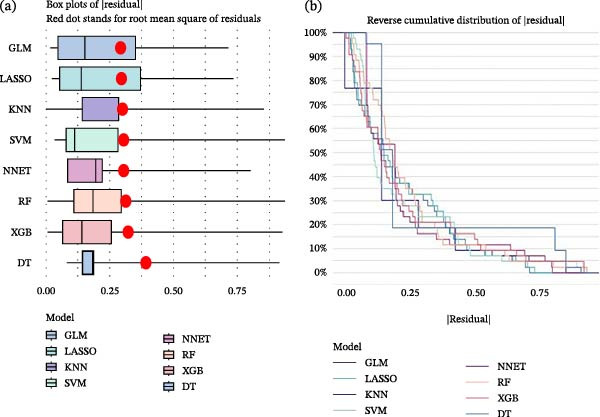

**Figure:**
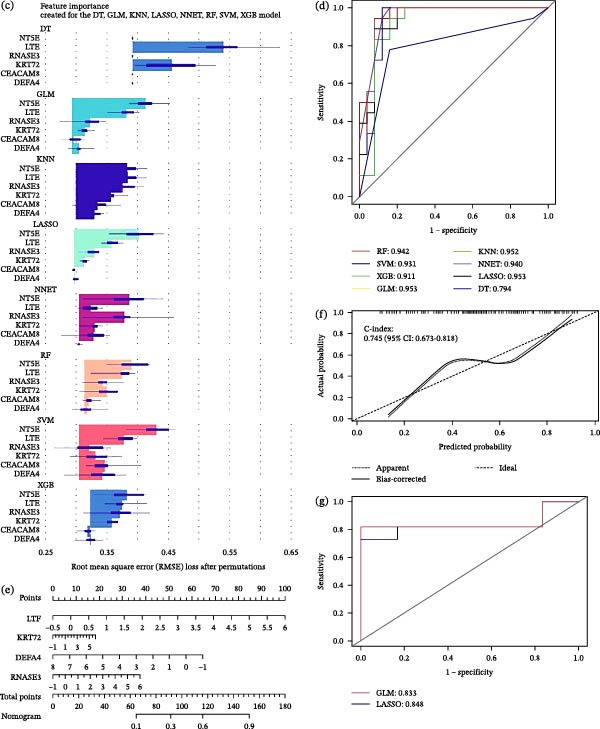

**Table 6:** Results of KNN model construction in OSA.

Variable	Permutation	Dropout_loss	Label
RNASE3	0	0.401894	KNN
CEACAM8	0	0.4129	KNN
KRT72	0	0.41378	KNN
LTF	0	0.414811	KNN
DEFA4	0	0.464039	KNN

**Table 7:** Results of LASSO model construction in sarcoidosis.

Variable	Permutation	Dropout_loss	Label
KRT72	0	0.313507	LASSO
RNASE3	0	0.329479	LASSO
LTF	0	0.35832	LASSO
NT5E	0	0.402745	LASSO

**Table 8:** Results of GLM model construction in sarcoidosis.

Variable	Permutation	Dropout_loss	Label
KRT72	0	0.314213	GLM
RNASE3	0	0.322425	GLM
LTF	0	0.3824	GLM
NT5E	0	0.412358	GLM
DEFA4	0	0.167823	GLM

### 3.6. Results of pQTL Colocalization Analysis

We conducted protein‐level colocalization analysis of four genes associated with both OSA and sarcoidosis. The results showed that the posterior probabilities for RNASE3 and LTF colocalizing in both OSA and sarcoidosis exceeded 70% (Figure 7b–d and Tables S5–S8), indicating a potential shared genetic basis. Although the posterior probability of KRT72 did not exceed 70%, the PPH3/PPH4 ratio was greater than 1—a relaxed criterion suggesting a potential genetic association between KRT72, OSA, and sarcoidosis [19]. Validation was not performed for DEFA4 due to the absence of corresponding data in the pQTL Consortium.

### 3.7. Results of Immune Microenvironment Immune Scoring

Three immune microenvironment scoring methods were applied to evaluate immune responses between the OSA and control groups as well as between the sarcoidosis and control groups. The results showed that immune scores differed significantly between sarcoidosis and healthy controls (Figure 4g). In contrast, among OSA and controls, only the immune score showed a statistically significant difference (Figure 4h). These findings further support the involvement of immune‐related mechanisms and immune cell dysregulation in both OSA and sarcoidosis.

### 3.8. Results of MR Analysis of Immune Cells and OSA and Sarcoidosis

Immune microenvironment analysis revealed differences in immune cell composition between the OSA group and the sarcoidosis group compared to controls, suggesting a potential involvement of immune cells in both diseases. Therefore, we conducted MR analysis using 731 immune cell phenotypes as exposures and OSA and sarcoidosis as outcomes. In the presence of heterogeneity, a random‐effect IVW model was used; otherwise, a fixed‐effect IVW model was used. Furthermore, FDR correction was applied to adjust the *p*‐values. In addition, MR‐Egger and Cochran *Q* test analyses were performed to assess horizontal diversity and heterogeneity, and the results are shown in Tables S9–S12.

Several immune cell phenotypes were identified as risk factors for OSA, including CD33dim HLA DR‐ AC(IVW OR = 1.024, 95% CI: 1.002–1.046, *p* = 0.0295), basophil AC(IVW OR = 1.023, 95% CI: 1.002–1.0454, *p* = 0.0331), CD28+ CD45RA+ CD8dim AC(IVW OR = 1.006, 95% CI: 1.001–1.011, *p* = 0.016), HLA DR on CD14^+^ CD16^+^ monocyte(IVW OR = 1.04, 95% CI: 1.002–1.080, *p* = 0.038) and CD8 on CD28+ CD45RA‐ CD8br(IVW OR = 1.102, 95% CI: 1.029–1.182, *p* = 0.006). In contrast, FSC‐A on plasmacytoid DC(IVW OR = 0.901, 95% CI: 0.819–0.991, *p* = 0.032) and CD20 on IgD+ CD38‐(IVW OR = 0.897, 95% CI: 0.814–0.989, *p* = 0.029) were protective against OSA.

In sarcoidosis, CD28 on CD28+ CD45RA+ CD8br (IVW OR = 1.3102, 95% CI: 1.117–1.1537, *p* = 0.0009) and CD16 on CD14^+^ CD16^+^ monocyte(IVW OR = 1.8253, 95% CI: 1.7128–1.9556, *p* = 0.0103) were identified as significant risk factors. In contrast, several immune phenotypes were found to be protective against sarcoidosis, including: HLA DR on CD14‐ CD16‐(IVW OR = 0.7838, 95% CI: 0.6474–0.9490, *p* = 0.0125), CD39+ CD8br %CD8br(IVW OR = 0.8099, 95% CI: 0.7073–0.9274, *p* = 0.0023), CD39+ CD8br AC (IVW OR = 0.8130, 95% CI: 0.7124–0.9277, *p* = 0.0021), CD20 on IgD+ CD38dim (*p* = 0.001, 95% CI: 0.5905–0.9490, IVW OR = 0.8719), HLA DR + NK %NK (IVW OR = 0.8408, 95% CI: 0.7130–0.9913, *p* = 0.039), CD39+ CD8br %T cell (IVW OR = 0.7999, 95% CI: 0.7130–0.9913, *p* = 0.0005) and IgD‐ CD27‐ %B cell(*p* = 0.046376, IVW OR = 0.844281, 95% CI: 0.7051–0.9074).

Although no identical immunophenotypes were found to be significantly associated with both OSA and sarcoidosis after FDR correction, our results identified two potential immunophenotypes of CD14^+^CD16^+^ monocytes that were associated with OSA and sarcoidosis, respectively (Table S13).

### 3.9. Results of the Immune Infiltration Analysis of DEGs

We also performed immune infiltration analysis, focusing on the correlation between DEGs and immune cells. The results are shown in Figure 4e,f. Monocytes showed a significant positive correlation with DEFA4, RNASE3, CEACAM8, and LTF in both OSA and sarcoidosis samples (*p*  < 0.05). Follicular helper T cells were significantly negatively correlated with DEFA4, RNASE3, CEACAM8, and LTF in both OSA and sarcoidosis samples (*p*  < 0.05).

### 3.10. Results of Clustering of DEGs and Analyses Between DEGs Clusters

Based on the expression profiles of DEGs, we classified both OSA and sarcoidosis samples into two clusters: C1 and C2 (Figure 8a,b, Table S14, Figure 9a,b, and Table S15). We also performed differential expression analysis between C1 and C2 clusters in both OSA and sarcoidosis samples. In sarcoidosis, CEACAM8, DEFA4, and RNASE3 were significantly upregulated in C2, while NT5E and KRT72 were significantly upregulated in C1 (Figure 9c). In OSA samples, DEFA4, LTF, RNASE3, CEACAM8, and NT5E showed significant upregulation in C2 (Figure 8c). Immune infiltration analysis based on DEG‐derived clustering revealed distinct immune cell distributions. In OSA samples, monocytes, M2 macrophages, Tregs, and memory B cells were significantly upregulated in the C2 cluster, whereas naïve B cells, plasma cells, resting CD4+ memory T cells, and activated CD4+ memory T cells were significantly upregulated in C1 (Figure 8d,e). In sarcoidosis samples, C1 showed significant upregulation of naïve CD4+ T cells, resting CD4+ memory T cells, and follicular helper T cells, while C2 was enriched for monocytes, M0 macrophages, resting NK cells, resting mast cells, eosinophils, and neutrophils (Figure 9d,e). In addition, GSVA analysis identified several GO‐ and KEGG‐enriched pathways in both OSA and sarcoidosis, which exhibited statistically significant expression differences between the C1 and C2 clusters (Figure 8f,g and Figure 9f,g).

Figure 8The first clustering and correlation analysis of OSA samples. (a) Consensus matrix heat map of DEGs clustering for OSA samples; (b) cumulative distribution function; (c) heat map of DEGs expression between DEG clusters; (d) heat map of immune cell ratios between DEG clusters; (e) box plot of immune cell fraction between DEGs clusters; (f, g) bar plot of GO terms and KEGG terms of GSVA between DEGs clusters.

**Figure:**
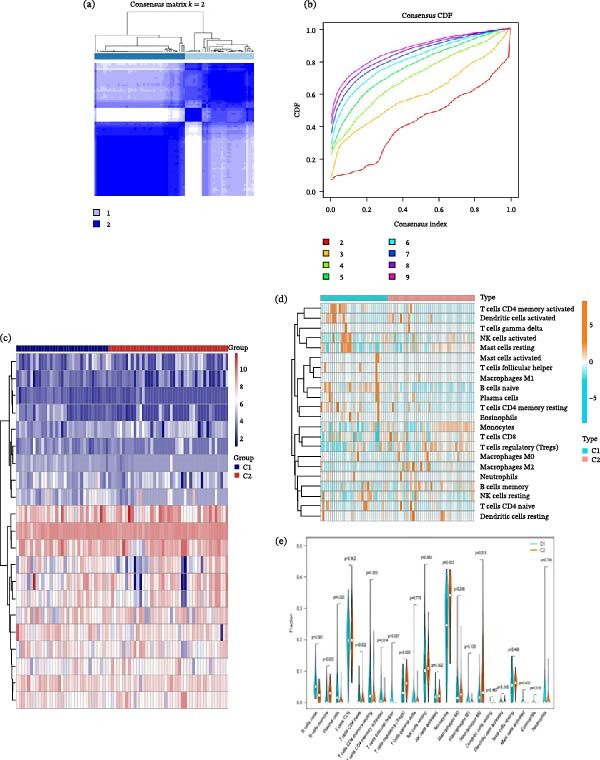

**Figure:**
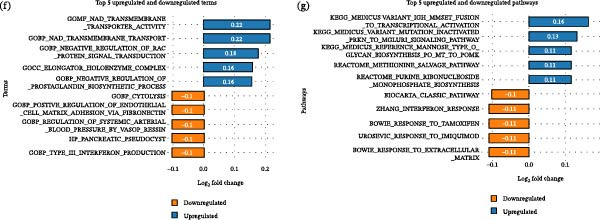

Figure 9The first clustering and correlation analysis of sarcoidosis samples. (a) Consensus matrix heat map of DEGs clustering for sarcoidosis samples; (b) cumulative distribution function; (c) heat map of DEGs expression between DEG clusters; (d) heat map of immune cell ratios between DEG clusters; (e) box plot of immune cell fraction between DEGs clusters; (f, g) bar plot of GO terms and KEGG terms of GSVA between DEGs clusters; (h) the DEAG were overlapped by Venn diagram in OSA and sarcoidosis.

**Figure:**
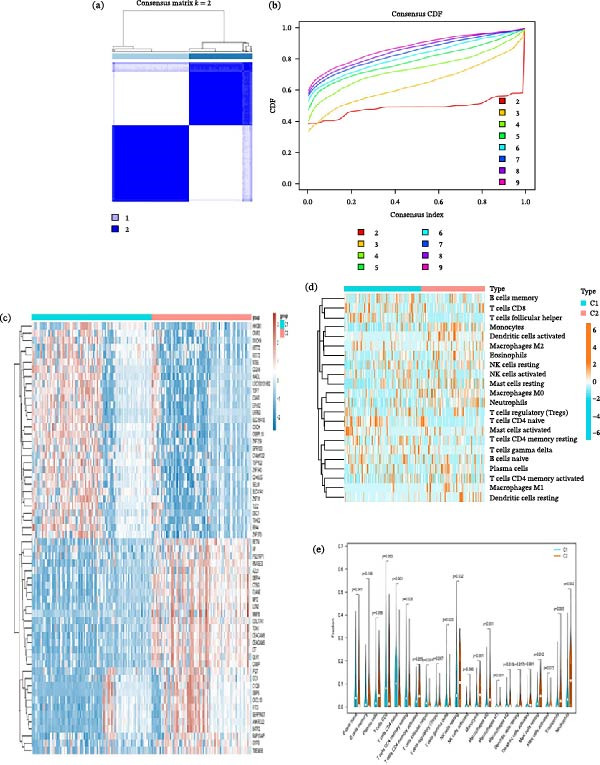

**Figure:**
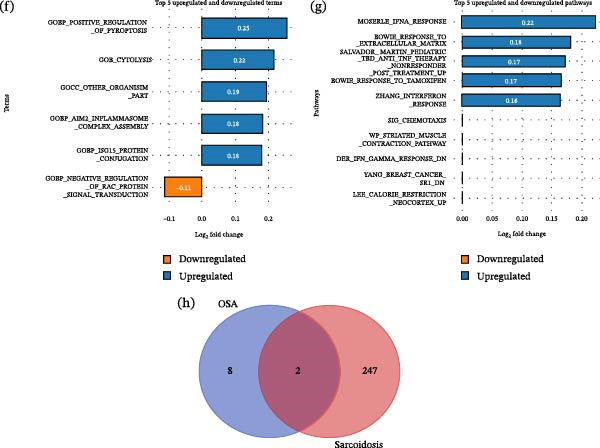

### 3.11. Results of Clustering of DEAGs and Analyses Between DEAGs Clusters

**Figure 10:**
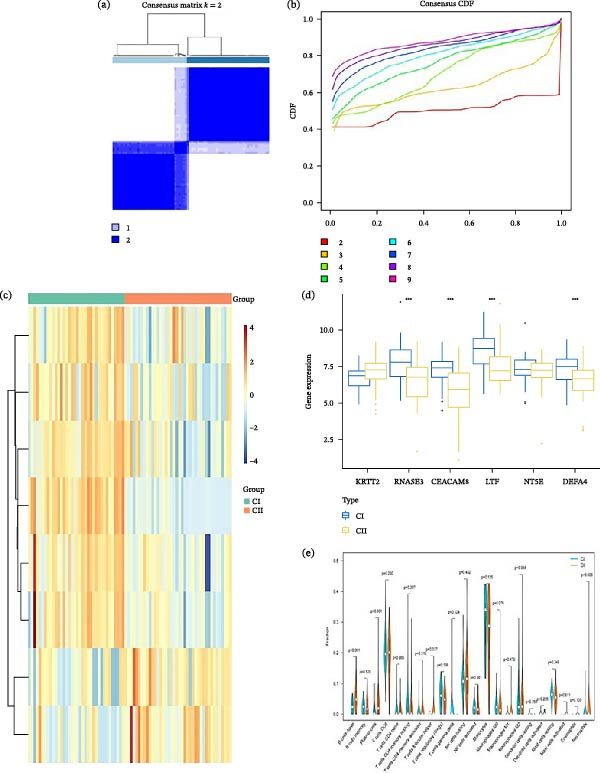
The second clustering and correlation analysis of OSA samples. (a) Consensus matrix heat map of DEAG clustering for OSA samples; (b) cumulative distribution function; (c) heat map of DEGs expression between DEAG clusters; (d) box plot of expression difference analysis between DEAG clusters (Wilcoxon rank‐sum test); (e) box plot of immune cell fraction between DEAG clusters.

**Figure 11:**
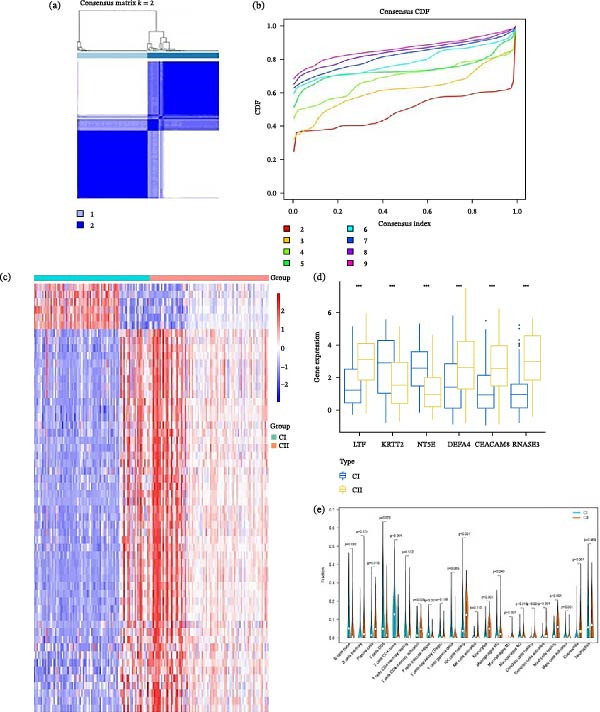
The second clustering and correlation analysis of sarcoidosis samples. (a) Consensus matrix heat map of DEAG clustering for sarcoidosis samples; (b) cumulative distribution function; (c) heat map of DEGs expression between DEAG clusters; (d) box plot of expression difference analysis between DEAG clusters (Wilcoxon rank‐sum test); (e) box plot of immune cell fraction between DEAG clusters.

Based on differential expression analyses of C1 and C2 clusters in both OSA and sarcoidosis samples, intersecting the resulting DEAGs using Venn diagrams identified two DEAGs associated with the co‐occurrence of OSA and sarcoidosis (Table S16, S17, Figure 9h). According to DEAG, OSA samples were clustered into CI and CII (Figure 10a,b and Table S18), and sarcoidosis samples were also clustered into CI and CII (Figure 11a,b and Table S19). Subsequently, we performed differential expression analysis of DEGs based on DEAG clustering. In OSA samples, NT5E, KRT72, DEFA4, CEACAM8, RNASE3, MMP8, and IDO1 were all upregulated in the CI cluster (Figure 10c). In sarcoidosis samples, NT5E was significantly upregulated in CI, while DEFA4, CEACAM8, RNASE3, MMP8, and IDO1 were significantly upregulated in CII (Figure 11c). We also used box plots to visualize the expression of DEGs across DEAG‐defined groups. In OSA samples, RNASE3, LTF, CEACAM8, and DEFA4 showed higher expression in the CI cluster (Figure 10d). In sarcoidosis samples, DEFA4, CEACAM8, LTF, and RNASE3 were more highly expressed in the CII cluster, whereas KRT72 and NT5E showed higher expression in the CI cluster (Figure 11d). In addition, immune infiltration analysis based on DEAG clustering revealed that in OSA samples, naive B cells and plasma cells were significantly upregulated in the CII cluster (Figure 10e). In sarcoidosis samples, monocytes, M1 macrophages, resting NK cells, eosinophils, and resting mast cells were significantly upregulated in the CII cluster, whereas naive CD4 T cells were significantly upregulated in the CI cluster (Figure 11e).

### 3.12. Results of DEGs Scoring and Related Analysis

**Figure 12:**
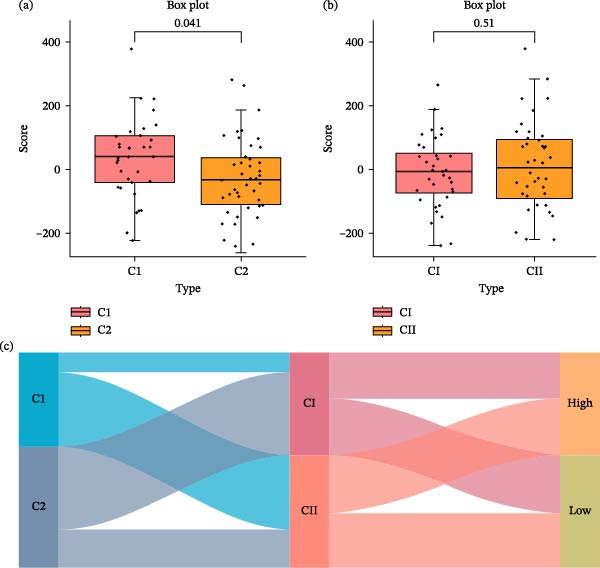
Box plots and alluvial plots of DEGs scores in OSA samples. (a) Box plot of different expression analysis of DEGs score between DEGs cluster; (b) box plot of different expression analysis of DEGs score between DEAG clusters; (c) alluvial plot of the correspondence of the different clustered samples in obstructive sleep apnea.

**Figure 13:**
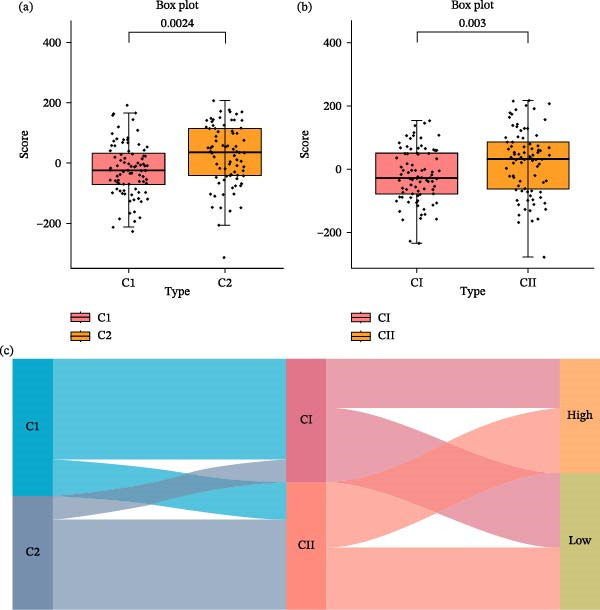
Box plots and alluvial plots of DEGs scores in sarcoidosis samples. (a) Box plot of different expression analysis of DEGs score between DEGs clusters; (b) box plot of different expression analysis of DEGs score between DEAG clusters; (c) alluvial plot of the correspondence of the different clustered samples in sarcoidosis.

The PCA method was used to construct a DEGs score. The results showed that in OSA samples, the C1 score was significantly higher, whereas the C2 score was significantly lower. In the DEG cluster, C1 scores were lower, and C2 scores were higher; however, this difference lacked statistical significance (Figure 12a,b). The constructed alluvial diagram shows that C1 primarily corresponds to CII and C2 primarily corresponds to CI, which aligns with our previous analytical results (Figure 12c). In the sarcoidosis samples, the C2 scores were significantly higher, while the C1 scores were lower. In the DEAG clustering, CII scores were significantly higher, while CI scores were lower (Figure 13a,b). The alluvial plot further illustrates that CI primarily corresponds to C1, whereas C2 mainly corresponds to CII (Figure 13c).

## 4. Discussion

Several studies have identified a high prevalence of OSA among patients with sarcoidosis. However, the small sample sizes and high heterogeneity of these studies limit the generalizability of their findings [3, 6, 10]. The genetic association between OSA and sarcoidosis remains challenging to evaluate, largely owing to confounding biases inherent in observational studies. On the other hand, the sample sizes in these studies were relatively small. Our MR analysis revealed that sarcoidosis does not genetically increase the risk of OSA. This may be explained by mechanical factors—such as lung parenchymal involvement and structural alterations of the airway that occur over the long disease course of sarcoidosis—rather than by genetic predisposition. OSA was identified as a risk factor for sarcoidosis through large‐scale GWAS data analysis, with meta‐analysis confirming this association. OSA is associated with several inflammatory factors, which are also important mediators of granuloma formation [20]. In addition, a study found that hypoxia can promote the development of sarcoidosis through inflammatory and fibrotic responses mediated by monocyte–macrophage activity [21]. One of the main characteristics of OSA is intermittent hypoxia. This could provide a possible explanation for the association between them. Furthermore, a Swedish nested case–control study reported that elevated inflammatory markers may precede the diagnosis of sarcoidosis [22]. In particular, IL‐6 is associated with the severity of OSA [23]. It promotes Th1/Th17 cell differentiation and granuloma formation, and its levels are known to increase even in healthy individuals in response to poor sleep quality and sleep fragmentation [24, 25]. Given these findings, it is biologically plausible that OSA may exacerbate immune activation in susceptible individuals, thereby potentially enhancing the immunopathogenesis of sarcoidosis. This hypothesis is further supported by evidence showing that OSA is associated with an increased risk of several autoimmune diseases, including rheumatoid arthritis, systemic lupus erythematosus (SLE), and ankylosing spondylitis [26].

### 4.1. Mechanism Analysis of the Hub Genes

#### 4.1.1. DEFA4

DEFA4 is a member of the alpha‐defensin class of short antimicrobial peptides, predominantly found in neutrophils, and plays critical roles in inflammatory cell recruitment and pathogen clearance [27]. Moreover, it is abundant in the bronchial epithelium [28]. In a study of nonobese OSA patients, increased AHI was positively correlated with elevated transcriptional expression of DEFA4, indicating its role in neutrophil‐mediated inflammation [29]. Additionally, DEFA4 contributes to neutrophil maturation and the formation of neutrophil extracellular traps (NETs) and has been observed to be elevated in patients with SLE [30, 31]. Two studies identified elevated neutrophil proportions in bronchoalveolar lavage fluid (BALF) during the disease course of sarcoidosis [32, 33]. Therefore, we hypothesized that DEFA4 contributes to the pathogenesis of OSA comorbid with sarcoidosis mediated by neutrophil activity.

#### 4.1.2. LTF

LTF, a member of the transferrin family, is an iron‐binding protein [34]. Emerging evidence suggests that LTF may modulate the regulation of neural activity [35]. Furthermore, sleep disruption and shortened sleep duration represent hallmark clinical features in patients with OSA. In a rodent study, exogenous LTF supplementation was shown to ameliorate sleep disruption in a rat model [36]. Moreover, in a randomized controlled trial, it was observed that administering LTF to children alleviated sleep deprivation [37]. In addition, the NF‐κB factor is involved in the development of systemic inflammation in OSA [38]. On the other hand, sarcoidosis has been associated with immune responses marked by NF‐κB activation [39]. According to a meta‐analysis, LTF may serve as a promising therapeutic target by modulating the NF‐κB signaling pathway [40]. Therefore, LTF represents a promising therapeutic target for intervention in OSA and sarcoidosis.

#### 4.1.3. RNASE3

RNASE 3, also known as eosinophil cationic protein (ECP), is a cationic protein implicated in immune responses [41]. Mouse experiments revealed elevated RNASE3 levels in mice exposed to chronic intermittent hypoxia (CIH), indicating a role for macrophages in mediating CIH‐induced inflammation [42]. Furthermore, macrophages are involved in the formation of granuloma mediators [43]. Previous studies have established an association between RNASE3 and IPF [44]. In particular, some forms of sarcoidosis share some common pathological features with IPF, such as end‐stage sarcoidosis [45]. Current evidence indicates that pulmonary fibrosis is driven by RNASE3‐mediated cytotoxicity, which directly destroys lung epithelial cells [46]. Given the high prevalence of OSA in IPF, we hypothesize that RNASE3 may serve as a shared molecular mediator linking OSA to sarcoidosis and pulmonary fibrosis [47].

#### 4.1.4. KRT72

KRT72, a member of the keratin family, primarily contributes to the structural integrity of the cellular cytoskeleton [48]. Prior studies have identified KRT72 as a potential biomarker in prostate cancer, but its role in OSA and sarcoidosis remains uncharacterized [49]. Our pathway analyses suggest that KRT72 may be involved in autophagy and DNA methylation repair mechanisms.

In summary, we found that several hub genes have been previously confirmed to be related to OSA or sarcoidosis, with underlying mechanisms involving not only the promotion of inflammation and immune responses but also the regulation of neural activity, cytotoxicity, and remodeling of the immune microenvironment.

### 4.2. Mechanism Analysis of Immune Cells and Pathways Related to Associated Genes

We performed pathway and functional enrichment analyses of DEGs and conducted MR analysis to assess the causal relationships between 731 immune cell traits and OSA/sarcoidosis. Pathway and enrichment analysis showed that DEGs associated with OSA and sarcoidosis were enriched in immune response, inflammation, etc. The CD14^+^CD16^+^ monocyte subpopulation has been implicated in proinflammatory responses across diverse disease contexts [50]. Our MR analysis demonstrated a causal relationship between the CD14^+^CD16^+^ monocyte subpopulation and both OSA and sarcoidosis. In some severely obese patients, OSA is considered a key contributor to the increase in CD14^+^CD16^+^ monocytes [51]. Another study found that CD14^+^CD16^+^ monocytes were elevated in OSA patients, regardless of body weight [52]. In addition, CD14^+^CD16^+^ monocytes are recognized as sensitive biomarkers of active sarcoidosis. On the one hand, CD14^+^CD16^+^ monocyte subsets contribute to the development of sarcoidosis by infiltrating inflammatory mediators [53]. On the other hand, these subsets may also promote the onset and progression of OSA by releasing various inflammatory mediators [54].

We developed hub gene‐based nomograms to assess the risk of OSA‐sarcoidosis comorbidity. Furthermore, two cluster analyses were performed for OSA combined with sarcoidosis, and DEG scores were constructed separately. We also performed single‐gene pathway analysis on hub genes, indicating that hub genes are associated with pathways such as oxidative stress, remodeling of the immune microenvironment, proinflammation, autophagy, and immune response. At the same time, the direct correspondence between DEG expression levels and immune cells was observed, reinforcing the reliability of our findings.

### 4.3. Strengths and Limitations of This Study

Compared to traditional observational studies, MR analysis can substantially reduce the influence of confounding bias. To enhance the credibility of our results, we performed colocalization analysis to link MR findings with GEO transcriptomic data. We integrated multiple GEO datasets, identified DEGs among the candidate genes highlighted by colocalization analysis, and conducted two consensus clustering analyses based on DEG expression profiles. Furthermore, external pQTL validation supplemented our findings. This integrative approach enhances the accuracy and comprehensiveness of our study.

Our study also has certain limitations. First, some hub genes lack cis‐pQTL data, preventing external validation. Second, although we conducted internal and external validation, as well as two cluster analyses, the data were obtained from a public database and may be subject to limitations such as data bias and a small sample size. Although this study provides insights based on transcriptomic analysis, the findings have not been validated at the proteomic or functional level. Further studies using experimental approaches, such as immunohistochemistry and western blotting, are required to confirm the expression and biological roles of the identified targets. Finally, data based on GWAS summary statistics can only reflect the genetic association between OSA and sarcoidosis. To enhance integration with clinical practice, future research should incorporate relevant clinical data with genomics, further emphasizing the importance of combining clinical application with innovation [55].

## 5. Conclusion

This study first established a causal relationship between OSA and sarcoidosis through MR analysis. Subsequently, colocalization analysis and bioinformatics approaches were employed to investigate their genetic association. Using data from the GEO database, six DEGs implicated in the shared genetic background of OSA and sarcoidosis were identified. These hub genes may influence the onset, progression, and prognosis of sarcoidosis combined with OSA through mechanisms such as immune regulation, inflammation, DNA repair and methylation, apoptosis, immune microenvironment remodeling, autophagy, and neural activity regulation. Given the methodological rigor of this study, the findings may serve as a valuable reference for clinical practice and future research.

## Author Contributions

**Yi Li, Chengdian Lan, Haiyan Lei, and Zhi Lyu**: visualization, validation, supervision, software, resources, project administration, methodology, investigation, formal analysis, data curation, conceptualization, writing – original draft. **Danxia Lin and Jiali Shen**: visualization, validation, supervision, software, resources, project administration, methodology, investigation, formal analysis, data curation, conceptualization. **Hongzhan Jiang, Yulin Wang, and Congying Lu**: visualization, validation, resources, project administration, methodology, investigation, formal analysis, data curation, conceptualization. **Yao Liu**: visualization, validation, supervision, software, resources, project administration, methodology, investigation, funding acquisition, formal analysis, data curation, conceptualization, writing – review and editing.

## Funding

This work was supported by the Chronic Obstructive Pulmonary Disease Screening and Respiratory Rehabilitation Intervention in Haicang District of Xiamen (Grant KZHC202102).

## Disclosure

All authors read and approved the final manuscript.

## Ethics Statement

The analyses for this study were based on publicly available summary datasets, and no additional ethics approval or consent was needed.

## Consent

The authors have nothing to report.

## Conflicts of Interest

The authors declare no conflicts of interest.

## Supporting Information

Additional supporting information can be found online in the Supporting Information section.

## Supporting information


**Supporting Information** Table S1: Information of genetic instrumental variants of OSA in MR analysis. Table S2: Information of genetic instrumental variants of sarcoidosis in MR analysis. Table S3: Information of genetic instrumental variants of OSA in repeat MR analysis. Table S4: Information of genetic instrumental variants of sarcoidosis in repeat MR analysis. Table S5: Results of colocalization analysis about OSA and LTF. Table S6: Results of colocalization analysis about OSA and RNASE3. Table S7: Results of colocalization analysis about sarcoidosis and LTF. Table S8: Results of colocalization analysis about sarcoidosis and RNASE3. Table S9: MR results of OSA and immune cell phenotypes. Table S10: Pleiotropy and heterogeneity results of immune cells and OSA. Table S11: MR results of sarcoidosis and immune cell phenotypes. Table S12: Pleiotropy and heterogeneity results of immune cells and sarcoidosis. Table S13: MR analysis of immune cell phenotypes in sarcoidosis and OSA. Table S14: Results of DEGs clusters in OSA. Table S15: Results of DEGs clusters in sarcoidosis. Table S16: Results of differential expression analysis between DEGs clusters in sarcoidosis. Table S17: Results of differential expression analysis between DEGs clusters in OSA. Table S18: Results of DEAG clusters in OSA. Table S19: Results of DEAG clusters in sarcoidosis.

## Data Availability

All data generated are included in this published article and its Supporting Information files.
