# Supplementary material for: Integrated Bioinformatics Methods Were Employed to Investigate Potential Molecular Links Between Obstructive Sleep Apnea and Sarcoidosis
Source: Mediators Inflamm. 2026 Jun 18;2026:7909167. doi: 10.1155/mi/7909167 (PMC13277778; doi:10.1155/mi/7909167)
Supplement: Supplementary file 1 — Supporting Information Table S1: Information of genetic instrumental variants of OSA in MR analysis. Table S2: Information of genetic instrumental variants of sarcoidosis in MR analysis. Table S3: Information of genetic instrumental variants of OSA in repeat MR analysis. Table S4: Information of genetic instrumental variants of sarcoidosis in repeat MR analysis. Table S5: Results of colocalization analysis about OSA and LTF. Table S6: Results of colocalization analysis about OSA and RNASE3. Table S7: Results of colocalization analysis about sarcoidosis and LTF. Table S8: Results of colocalization analysis about sarcoidosis and RNASE3. Table S9: MR results of OSA and immune cell phenotypes. Table S10: Pleiotropy and heterogeneity results of immune cells and OSA. Table S11: MR results of sarcoidosis and immune cell phenotypes. Table S12: Pleiotropy and heterogeneity results of immune cells and sarcoidosis. Table S13: MR analysis of immune cell phenotypes in sarcoidosis and OSA. Table S14: Results of DEGs clusters in OSA. Table S15: Results of DEGs clusters in sarcoidosis. Table S16: Results of differential expression analysis between DEGs clusters in sarcoidosis. Table S17: Results of differential expression analysis between DEGs clusters in OSA. Table S18: Results of DEAG clusters in OSA. Table S19: Results of DEAG clusters in sarcoidosis. [file MI-2026-7909167-s001.doc]

Supplementary Materials for

**Integrated bioinformatics methods were employed to investigate potential molecular links between obstructive sleep apnea and sarcoidosis.**

**Supplementary Table**

Table S1. Information of genetic instrumental variants of OSA in MR analysis.

| SNP | effect_allele | other_allele | beta | eaf | chr | pos | pval | se | F |
| --- | --- | --- | --- | --- | --- | --- | --- | --- | --- |
| rs10507084 | T | C | 0.1076 | 0.1794 | 12 | 97753152 | 4.07E-11 | 0.0163 | 43.5758 |
| rs10928560 | T | C | -0.0874 | 0.1948 | 2 | 1.37E+08 | 3.17E-08 | 0.0158 | 30.59882 |
| rs142006783 | C | T | 0.1781 | 0.03779 | 16 | 74651733 | 4.95E-08 | 0.0327 | 29.66391 |
| rs4837016 | A | G | -0.0697 | 0.4662 | 9 | 1.28E+08 | 2.3E-08 | 0.0125 | 31.09149 |
| rs9937053 | A | G | 0.1021 | 0.4299 | 16 | 53799507 | 4.03E-16 | 0.0125 | 66.71561 |

Table S2. Information of genetic instrumental variants of sarcoidosis in MR analysis.

| SNP | effect_allele | other_allele | beta | eaf | chr | pos | se | pval | F |
| --- | --- | --- | --- | --- | --- | --- | --- | --- | --- |
| rs12563505 | T | C | 0.703 | 0.0327 | 1 | 67598170 | 0.1101 | 1.68E-10 | 40.76939 |
| rs2245168 | C | T | -0.27 | 0.484242 | 10 | 81921918 | 0.033 | 2.93E-16 | 66.94187 |
| rs28688207 | C | T | 0.7435 | 0.073889 | 6 | 32628660 | 0.0927 | 1.08E-15 | 64.3281 |
| rs3093958 | G | A | 0.3229 | 0.129262 | 6 | 31410521 | 0.0565 | 1.07E-08 | 32.6616 |
| rs72928925 | C | A | 0.2519 | 0.143013 | 2 | 2.04E+08 | 0.0436 | 7.77E-09 | 33.37963 |

Table S3. Information of genetic instrumental variants of OSA in repeat MR analysis.

| SNP | effect_allele | other_allele | beta | eaf | chr | pos | se | pval | F |
| --- | --- | --- | --- | --- | --- | --- | --- | --- | --- |
| rs10507084 | T | C | 0.1027 | 0.165277 | 12 | 97753152 | 0.0189 | 5.51E-08 | 29.52686 |
| rs10906985 | A | G | 0.066 | 0.377922 | 15 | 83569076 | 0.013 | 4.05E-07 | 25.77515 |
| rs11075985 | A | C | 0.1036 | 0.372052 | 16 | 53805207 | 0.013 | 1.99E-15 | 63.50864 |
| rs114983649 | A | C | 0.1542 | 0.042422 | 2 | 2.09E+08 | 0.0303 | 3.66E-07 | 25.89903 |
| rs116182122 | G | T | 0.3093 | 0.012658 | 1 | 15005016 | 0.0598 | 2.27E-07 | 26.75207 |
| rs11774552 | T | C | -0.0662 | 0.413919 | 8 | 10515679 | 0.0132 | 5.05E-07 | 25.15174 |
| rs142416325 | A | G | -0.3465 | 0.012233 | 5 | 1.8E+08 | 0.0667 | 2.09E-07 | 26.98701 |
| rs145058358 | C | A | 0.1578 | 0.055625 | 3 | 25376953 | 0.0315 | 5.58E-07 | 25.09533 |
| rs17212403 | A | G | 0.0915 | 0.129002 | 9 | 1.26E+08 | 0.0186 | 8.95E-07 | 24.20005 |
| rs17513135 | T | C | 0.0768 | 0.206307 | 1 | 40035686 | 0.0155 | 7.37E-07 | 24.55043 |
| rs17547430 | G | A | 0.1268 | 0.059807 | 7 | 30189348 | 0.0255 | 6.36E-07 | 24.72624 |
| rs181154734 | A | G | 0.406 | 0.005555 | 10 | 35687024 | 0.0791 | 2.83E-07 | 26.34505 |
| rs1956376 | T | C | 0.069 | 0.435307 | 14 | 29064053 | 0.0132 | 1.59E-07 | 27.32438 |
| rs2277339 | G | T | -0.1037 | 0.135468 | 12 | 57146069 | 0.0197 | 1.49E-07 | 27.70927 |
| rs9318788 | G | A | 1.4733 | 0.001113 | 13 | 82181923 | 0.3002 | 9.21E-07 | 24.0858 |

Table S4. Information of genetic instrumental variants of sarcoidosis in repeat MR analysis.

| SNP | effect_allele | other_allele | beta | eaf | chr | pos | se | pval | F |
| --- | --- | --- | --- | --- | --- | --- | --- | --- | --- |
| rs12563505 | T | C | 0.703 | 0.0327 | 1 | 67598170 | 0.1101 | 1.68E-10 | 40.76956 |
| rs2236557 | A | G | -0.27 | 0.484131 | 10 | 81923961 | 0.033 | 2.9E-16 | 66.94215 |
| rs2361149 | C | G | 0.1824 | 0.253034 | 1 | 25194060 | 0.0372 | 9.63E-07 | 24.04162 |
| rs28688207 | C | T | 0.7435 | 0.073889 | 6 | 32628660 | 0.0927 | 1.08E-15 | 64.32836 |
| rs28810078 | A | G | 0.6851 | 0.018433 | 10 | 1.04E+08 | 0.1396 | 9.25E-07 | 24.08447 |
| rs36051895 | T | G | 0.1844 | 0.306954 | 9 | 4981866 | 0.0351 | 1.49E-07 | 27.59991 |
| rs554103485 | G | A | 1.3448 | 0.057966 | 4 | 1.82E+08 | 0.2593 | 2.15E-07 | 26.8974 |
| rs663743 | A | G | -0.1773 | 0.312813 | 11 | 64107735 | 0.0346 | 3.01E-07 | 26.25822 |
| rs72928925 | C | A | 0.2519 | 0.143013 | 2 | 2.04E+08 | 0.0436 | 7.77E-09 | 33.37977 |
| rs7748141 | C | T | 0.3294 | 0.129118 | 6 | 31288877 | 0.0568 | 6.45E-09 | 33.63183 |
| rs77527755 | T | G | -0.5948 | 0.113233 | 6 | 32525779 | 0.0856 | 3.59E-12 | 48.28302 |

Table S5. Results of colocalization analysis about OSA and LTF

| results.snp | results.pvalues.df1 | results.MAF.df1 | results.N.df1 | results.V.df1 | results.z.df1 | results.r.df1 | results.lABF.df1 | results.pvalues.df2 | results.MAF.df2 | results.N.df2 | results.V.df2 | results.z.df2 | results.r.df2 | results.lABF.df2 | results.internal.sum.lABF | results.SNP.PP.H4 | chr | pos |
| --- | --- | --- | --- | --- | --- | --- | --- | --- | --- | --- | --- | --- | --- | --- | --- | --- | --- | --- |
| rs61740470 | 0.709856 | 0.00814 | 453733 | 0.001287 | 0.372049 | 0.968819 | -1.66691 | 6.5E-133 | 0.00814 | 35343 | 0.001752 | 24.53372 | 0.92775 | 277.8941 | 276.2272 | 1 | 3 | 46450495 |
| rs181225830 | 0.452827 | 0.01141 | 453733 | 0.000921 | 0.75071 | 0.977482 | -1.62128 | 1.45E-68 | 0.01141 | 35344 | 0.001254 | 17.49947 | 0.947203 | 143.561 | 141.9397 | 1 | 3 | 46281089 |
| rs12491948 | 0.636455 | 0.01192 | 453733 | 0.000883 | 0.472661 | 0.978414 | -1.80855 | 4.19E-42 | 0.01192 | 35342 | 0.001201 | 13.59672 | 0.94932 | 86.25962 | 84.45107 | 1 | 3 | 46871689 |
| rs185998157 | 0.969948 | 0.00418 | 453733 | 0.002497 | 0.037674 | 0.941241 | -1.41649 | 2.35E-18 | 0.00418 | 35346 | 0.003398 | 8.73904 | 0.86878 | 32.15928 | 30.74279 | 1 | 3 | 45599526 |
| rs4683188 | 0.002472 | 0.00872 | 453733 | 0.001202 | 3.026788 | 0.970816 | 2.679978 | 3.59E-30 | 0.00872 | 35345 | 0.001637 | 11.41324 | 0.932196 | 59.36932 | 62.0493 | 1 | 3 | 46150577 |
| rs143839225 | 0.324953 | 0.01101 | 453733 | 0.000955 | 0.984331 | 0.976692 | -1.40632 | 1.82E-49 | 0.01101 | 35343 | 0.001299 | 14.78546 | 0.945409 | 101.8839 | 100.4776 | 1 | 3 | 46731886 |
| rs2742400 | 0.151935 | 0.00663 | 453733 | 0.001578 | 1.43273 | 0.962042 | -0.64824 | 7.33E-21 | 0.00663 | 35346 | 0.002148 | 9.368903 | 0.912858 | 38.84358 | 38.19533 | 0.999995 | 3 | 45783419 |
| rs1457787216 | 0.138708 | 0.00325 | 453733 | 0.003209 | 1.480619 | 0.925741 | -0.28538 | 1.47E-39 | 0.00325 | 35343 | 0.004367 | 13.16091 | 0.837454 | 71.61915 | 71.33377 | 0.999993 | 3 | 47311509 |
| rs12491948 | 0.636455 | 0.01192 | 453733 | 0.000883 | 0.472661 | 0.978414 | -1.80855 | 4.19E-42 | 0.01192 | 35342 | 0.001201 | 13.59672 | 0.94932 | 86.25962 | 84.45107 | 0.957877 | 3 | 46919307 |

Table S6. Results of colocalization analysis about OSA and RNASE3

| results.snp | results.pvalues.df1 | results.MAF.df1 | results.N.df1 | results.V.df1 | results.z.df1 | results.r.df1 | results.lABF.df1 | results.pvalues.df2 | results.MAF.df2 | results.N.df2 | results.V.df2 | results.z.df2 | results.r.df2 | results.lABF.df2 | results.internal.sum.lABF | results.SNP.PP.H4 | chr | pos |
| --- | --- | --- | --- | --- | --- | --- | --- | --- | --- | --- | --- | --- | --- | --- | --- | --- | --- | --- |
| rs542955875 | 0.630278 | 0.00236 | 453733 | 0.004415 | 0.481336 | 0.900603 | -1.04999 | 2.4E-102 | 0.00236 | 35340 | 0.006009 | 21.48076 | 0.789219 | 181.3034 | 180.2534 | 1 | 14 | 21118717 |
| rs145837285 | 0.890144 | 0.05478 | 453733 | 0.000201 | 0.138122 | 0.995007 | -2.64032 | 4E-241 | 0.05478 | 35337 | 0.000273 | 33.16022 | 0.988001 | 540.9912 | 538.3509 | 1 | 14 | 20788017 |
| rs147307766 | 0.227806 | 0.03506 | 453733 | 0.000307 | 1.20603 | 0.992378 | -1.71662 | 1.4E-255 | 0.03506 | 35340 | 0.000418 | 34.14918 | 0.981752 | 570.4415 | 568.7249 | 1 | 14 | 21004856 |
| rs71416932 | 0.470028 | 0.00253 | 453733 | 0.004119 | 0.722433 | 0.906644 | -0.94907 | 1.25E-27 | 0.00253 | 35336 | 0.005607 | 10.89254 | 0.800512 | 46.68334 | 45.73427 | 1 | 14 | 20304752 |
| rs74481326 | 0.839926 | 0.00556 | 453733 | 0.00188 | 0.201988 | 0.955112 | -1.53231 | 1.29E-41 | 0.00556 | 35360 | 0.002557 | 13.51394 | 0.897937 | 80.85255 | 79.32024 | 1 | 14 | 21553624 |
| rs72659967 | 0.727133 | 0.01054 | 453733 | 0.000997 | 0.348942 | 0.975689 | -1.79902 | 6.42E-20 | 0.01054 | 35327 | 0.001357 | 9.137048 | 0.943114 | 37.93489 | 36.13587 | 0.999995 | 14 | 20583046 |
| rs752615489 | 0.015403 | 0.00307 | 453733 | 0.003396 | 2.422758 | 0.921741 | 1.431331 | 2.41E-30 | 0.00307 | 35346 | 0.004622 | 11.44805 | 0.829586 | 53.47709 | 54.90842 | 0.999993 | 14 | 21299039 |
| rs111634275 | 0.976808 | 0.01938 | 453733 | 0.000547 | 0.029071 | 0.986511 | -2.15253 | 1.28E-29 | 0.01938 | 35329 | 0.000745 | 11.30204 | 0.967962 | 60.10149 | 57.94896 | 0.999846 | 14 | 20746646 |
| rs79467620 | 0.221766 | 0.02106 | 453733 | 0.000504 | 1.221846 | 0.987553 | -1.45597 | 7.11E-14 | 0.02106 | 35357 | 0.000686 | 7.485768 | 0.970416 | 25.42921 | 23.97324 | 0.999818 | 14 | 21402738 |
| rs74740573 | 0.622375 | 0.01443 | 453733 | 0.000731 | 0.492487 | 0.982056 | -1.89116 | 1.96E-23 | 0.01443 | 35335 | 0.000995 | 9.975153 | 0.957652 | 46.06402 | 44.17285 | 0.998822 | 14 | 20702273 |
| rs55633173 | 0.15081 | 0.04556 | 453733 | 0.000239 | 1.436676 | 0.99406 | -1.53711 | 4.5E-214 | 0.04556 | 35341 | 0.000325 | 31.22747 | 0.985746 | 478.5021 | 476.965 | 0.949371 | 14 | 21033168 |

Table S7. Results of colocalization analysis about sarcoidosis and LTF

| results.snp | results.pvalues.df1 | results.MAF.df1 | results.N.df1 | results.V.df1 | results.z.df1 | results.r.df1 | results.lABF.df1 | results.pvalues.df2 | results.MAF.df2 | results.N.df2 | results.V.df2 | results.z.df2 | results.r.df2 | results.lABF.df2 | results.internal.sum.lABF | results.SNP.PP.H4 | chr | pos |
| --- | --- | --- | --- | --- | --- | --- | --- | --- | --- | --- | --- | --- | --- | --- | --- | --- | --- | --- |
| rs61740470 | 0.5958 | 0.00814 | 486673 | 0.036175 | 0.53045 | 0.525107 | -0.29846 | 6.5E-133 | 0.00814 | 35342 | 0.001752 | 24.53372 | 0.927748 | 277.8935 | 277.5951 | 1 | 3 | 46450495 |
| rs181225830 | 0.3277 | 0.01141 | 486673 | 0.025893 | 0.978757 | 0.607046 | -0.17627 | 1.45E-68 | 0.01141 | 35345 | 0.001254 | 17.49947 | 0.947204 | 143.5612 | 143.385 | 1 | 3 | 46281089 |
| rs12491948 | 0.6533 | 0.01192 | 486673 | 0.024798 | 0.449183 | 0.617304 | -0.41798 | 4.19E-42 | 0.01192 | 35341 | 0.001201 | 13.59672 | 0.949318 | 86.25951 | 85.84152 | 1 | 3 | 46919307 |
| rs143839225 | 0.6947 | 0.01101 | 486673 | 0.026823 | 0.392485 | 0.598598 | -0.41029 | 1.82E-49 | 0.01101 | 35343 | 0.001299 | 14.78546 | 0.945409 | 101.8839 | 101.4736 | 1 | 3 | 46731886 |
| rs185998157 | 0.01988 | 0.00418 | 486673 | 0.070166 | 2.328605 | 0.363089 | 0.758845 | 2.35E-18 | 0.00418 | 35345 | 0.003398 | 8.73904 | 0.868777 | 32.15917 | 32.91801 | 1 | 3 | 45599526 |
| rs2742400 | 0.3692 | 0.00663 | 486673 | 0.044346 | 0.897973 | 0.474235 | -0.13025 | 7.33E-21 | 0.00663 | 35346 | 0.002148 | 9.368903 | 0.912858 | 38.84358 | 38.71333 | 0.999956 | 3 | 45783419 |
| rs4683188 | 0.7727 | 0.00872 | 486673 | 0.033789 | 0.288845 | 0.542089 | -0.36793 | 3.59E-30 | 0.00872 | 35344 | 0.001637 | 11.41324 | 0.932194 | 59.36922 | 59.00129 | 0.995173 | 3 | 46150577 |
| rs180845328 | 0.1291 | 0.00343 | 486673 | 0.085444 | 1.517661 | 0.318868 | 0.175224 | 5.83E-22 | 0.00343 | 35346 | 0.004138 | 9.632454 | 0.844647 | 38.25389 | 38.42912 | 0.993803 | 3 | 45912181 |

Table S8. Results of colocalization analysis about sarcoidosis and RNASE3

| results.snp | results.pvalues.df1 | results.MAF.df1 | results.N.df1 | results.V.df1 | results.z.df1 | results.r.df1 | results.lABF.df1 | results.pvalues.df2 | results.MAF.df2 | results.N.df2 | results.V.df2 | results.z.df2 | results.r.df2 | results.lABF.df2 | results.internal.sum.lABF | results.SNP.PP.H4 | chr | pos |
| --- | --- | --- | --- | --- | --- | --- | --- | --- | --- | --- | --- | --- | --- | --- | --- | --- | --- | --- |
| rs542955875 | 0.2445 | 0.00236 | 486673 | 0.12405 | 1.163813 | 0.243828 | 0.025384 | 2.4E-102 | 0.00236 | 35341 | 0.006009 | 21.48076 | 0.789223 | 181.3045 | 181.3299 | 1 | 14 | 21118717 |
| rs145837285 | 0.5316 | 0.05478 | 486673 | 0.005641 | 0.625565 | 0.876412 | -0.87392 | 4E-241 | 0.05478 | 35338 | 0.000273 | 33.16022 | 0.988001 | 540.9914 | 540.1175 | 1 | 14 | 20788017 |
| rs147307766 | 0.4748 | 0.03506 | 486673 | 0.008633 | 0.714691 | 0.822484 | -0.65429 | 1.4E-255 | 0.03506 | 35341 | 0.000418 | 34.14918 | 0.981753 | 570.4418 | 569.7875 | 1 | 14 | 21004856 |
| rs74481326 | 0.4815 | 0.00556 | 486673 | 0.052824 | 0.703892 | 0.430924 | -0.17512 | 1.29E-41 | 0.00556 | 35365 | 0.002557 | 13.51394 | 0.89795 | 80.85367 | 80.67855 | 1 | 14 | 21553624 |
| rs188404029 | 0.6237 | 0.00419 | 486673 | 0.069999 | 0.490613 | 0.36364 | -0.18223 | 5.1E-46 | 0.00419 | 35338 | 0.003391 | 14.24096 | 0.869025 | 87.10496 | 86.92273 | 1 | 14 | 20762162 |
| rs72659967 | 0.3646 | 0.01054 | 486673 | 0.028006 | 0.906635 | 0.588187 | -0.20185 | 6.42E-20 | 0.01054 | 35333 | 0.001357 | 9.137048 | 0.943123 | 37.93519 | 37.73334 | 0.999995 | 14 | 20583046 |
| rs111634275 | 0.2379 | 0.01938 | 486673 | 0.015368 | 1.180252 | 0.722434 | -0.13767 | 1.28E-29 | 0.01938 | 35338 | 0.000745 | 11.30204 | 0.96797 | 60.10187 | 59.96419 | 0.999913 | 14 | 20746646 |
| rs752615489 | 0.8584 | 0.00307 | 486673 | 0.095429 | 0.178411 | 0.295358 | -0.17033 | 2.41E-30 | 0.00307 | 35359 | 0.00462 | 11.44805 | 0.829638 | 53.48035 | 53.31001 | 0.999893 | 14 | 21299039 |
| rs536786225 | 0.9659 | 0.02352 | 486673 | 0.012717 | 0.042751 | 0.75877 | -0.71031 | 3.41E-14 | 0.02352 | 35333 | 0.000616 | 7.581759 | 0.973345 | 26.16305 | 25.45274 | 0.999688 | 14 | 20550811 |
| rs79467620 | 0.315 | 0.02106 | 486673 | 0.014167 | 1.004786 | 0.738461 | -0.29781 | 7.11E-14 | 0.02106 | 35359 | 0.000686 | 7.485768 | 0.970418 | 25.42922 | 25.13141 | 0.9996 | 14 | 21402738 |
| rs74740573 | 0.8653 | 0.01443 | 486673 | 0.020537 | 0.169631 | 0.660757 | -0.53101 | 1.96E-23 | 0.01443 | 35333 | 0.000995 | 9.975153 | 0.957649 | 46.06393 | 45.53292 | 0.99959 | 14 | 20702273 |
| rs55633173 | 0.9791 | 0.04556 | 486673 | 0.006717 | 0.026197 | 0.856226 | -0.96946 | 4.5E-214 | 0.04556 | 35341 | 0.000325 | 31.22747 | 0.985746 | 478.5021 | 477.5327 | 0.843904 | 14 | 21033168 |

Table S9. MR results of OSA and immune cell phenotypes

| outcome | exposure | method | nsnp | pval | or | or_lci95 | or_uci95 |
| --- | --- | --- | --- | --- | --- | --- | --- |
| OSA | CD33dim HLA DR- AC | Inverse variance weighted | 3 | 0.029502 | 1.023893 | 1.002353 | 1.045895 |
| OSA | Basophil AC | Inverse variance weighted | 3 | 0.033145 | 1.023392 | 1.00185 | 1.045396 |
| OSA | CD28+ CD45RA+ CD8dim AC | Inverse variance weighted | 8 | 0.015514 | 1.006397 | 1.001213 | 1.011608 |
| OSA | CD20 on IgD+ CD38- | Inverse variance weighted | 4 | 0.028758 | 0.897231 | 0.814128 | 0.988817 |
| OSA | FSC-A on plasmacytoid DC | Inverse variance weighted | 4 | 0.032017 | 0.901033 | 0.819161 | 0.991088 |
| OSA | HLA DR on CD14+ CD16+ monocyte | Inverse variance weighted | 6 | 0.03777 | 1.039979 | 1.002217 | 1.079165 |
| OSA | CD8 on CD28+ CD45RA- CD8br | Inverse variance weighted | 3 | 0.005862 | 1.102488 | 1.028565 | 1.181724 |

Table S10. pleiotropy and heterogeneity results of immune cells and OSA

| outcome | exposure | egger_intercept | se | pval | Q | Q_df | Q_pval |
| --- | --- | --- | --- | --- | --- | --- | --- |
| Obstructive sleep apnoea | CD33dim HLA DR- AC | 0.006502004 | 0.02027897 | 0.802474432 | 1.982775 | 2 | 0.371061 |
| Obstructive sleep apnoea | CD28+ CD45RA+ CD8dim AC | -0.002418926 | 0.01117369 | 0.835783213 | 7.467821 | 7 | 0.381846 |
| Obstructive sleep apnoea | Basophil AC | 0.004569769 | 0.01889958 | 0.848969071 | 1.628798 | 2 | 0.442905 |
| Obstructive sleep apnoea | CD20 on IgD+ CD38- | 0.043185097 | 0.08220942 | 0.651797986 | 3.458133 | 3 | 0.326233 |
| Obstructive sleep apnoea | FSC-A on plasmacytoid DC | 0.014783198 | 0.02328363 | 0.590428298 | 1.139527 | 3 | 0.767542 |
| Obstructive sleep apnoea | HLA DR on CD14+ CD16+ monocyte | -0.029528559 | 0.02822097 | 0.354466521 | 4.656181 | 5 | 0.459263 |
| Obstructive sleep apnoea | CD8 on CD28+ CD45RA- CD8br | -0.020656459 | 0.02702054 | 0.584478677 | 0.58742 | 2 | 0.745493 |

Table S11. MR results of sarcoidosis and immune cell phenotypes

| outcome | exposure | method | nsnp | pval | or | or_lci95 | or_uci95 |
| --- | --- | --- | --- | --- | --- | --- | --- |
| Sarcoidosis | IgD- CD27- %B cell | Inverse variance weighted | 3 | 0.046376 | 0.844281 | 0.714748 | 0.997289 |
| Sarcoidosis | HLA DR+ NK %NK | Inverse variance weighted | 5 | 0.039096 | 0.840763 | 0.713041 | 0.991363 |
| Sarcoidosis | CD39+ CD8br %T cell | Inverse variance weighted | 7 | 0.000521 | 0.799904 | 0.705126 | 0.907422 |
| Sarcoidosis | CD39+ CD8br %CD8br | Inverse variance weighted | 7 | 0.002281 | 0.80988 | 0.707276 | 0.927368 |
| Sarcoidosis | CD39+ CD8br AC | Inverse variance weighted | 6 | 0.002115 | 0.812973 | 0.712413 | 0.927728 |
| Sarcoidosis | CD20 on IgD+ CD38dim | Inverse variance weighted | 8 | 0.000842 | 0.717584 | 0.590548 | 0.871946 |
| Sarcoidosis | CD28 on CD28+ CD45RA+ CD8br | Inverse variance weighted | 3 | 0.000919 | 1.310224 | 1.116733 | 1.537241 |
| Sarcoidosis | CD16 on CD14+ CD16+ monocyte | Inverse variance weighted | 5 | 0.010269 | 1.825343 | 1.712808 | 1.955644 |
| Sarcoidosis | HLA DR on CD14- CD16- | Inverse variance weighted | 5 | 0.012547 | 0.783832 | 0.647401 | 0.949014 |

Table S12. pleiotropy and heterogeneity results of immune cells and sarcoidosis

| outcome | exposure | egger_intercept | se | pval | Q | Q_df | Q_pval |
| --- | --- | --- | --- | --- | --- | --- | --- |
| Sarcoidosis | IgD- CD27- %B cell | 0.033815992 | 0.05785172 | 0.66324988 | 0.507128 | 2 | 0.77603 |
| Sarcoidosis | HLA DR+ NK %NK | 0.015213224 | 0.04718375 | 0.768293847 | 6.712362 | 4 | 0.151892 |
| Sarcoidosis | CD39+ CD8br %T cell | 0.009994288 | 0.05407304 | 0.860625995 | 4.094507 | 6 | 0.663889 |
| Sarcoidosis | CD39+ CD8br %CD8br | 0.003009806 | 0.05794786 | 0.960587803 | 3.615605 | 6 | 0.728531 |
| Sarcoidosis | CD39+ CD8br AC | -0.016839228 | 0.0563604 | 0.779988788 | 3.955532 | 5 | 0.555836 |
| Sarcoidosis | CD20 on IgD+ CD38dim | -0.005783962 | 0.12211948 | 0.963760951 | 14.08573 | 7 | 0.05 |
| Sarcoidosis | CD28 on CD28+ CD45RA+ CD8br | -0.064502992 | 0.23732588 | 0.831053711 | 1.684026 | 2 | 0.430842 |
| Sarcoidosis | CD16 on CD14+ CD16+ monocyte | -0.038585885 | 0.11896376 | 0.766972604 | 6.681954 | 4 | 0.153681 |
| Sarcoidosis | HLA DR on CD14- CD16- | -0.07256525 | 0.10291883 | 0.531577279 | 10.46988 | 4 | 0.053214 |

Table S13. MR analysis of immune cell phenotypes in sarcoidosis and OSA

| outcome | exposure | method | nsnp | pval | or | or_lci95 | or_uci95 |
| --- | --- | --- | --- | --- | --- | --- | --- |
| Sarcoidosis | CD16 on CD14+ CD16+ monocyte | Inverse variance weighted | 5 | 0.010269 | 1.825343 | 1.712808 | 1.955644 |
| OSA | HLA DR on CD14+ CD16+ monocyte | Inverse variance weighted | 6 | 0.03777 | 1.039979 | 1.002217 | 1.079165 |

Table S14. Results of DEGs clusters in OSA

| ID | DEGs cluster |
| --- | --- |
| GSM4037879 | C1 |
| GSM4037880 | C1 |
| GSM4037881 | C1 |
| GSM4037883 | C1 |
| GSM4037884 | C1 |
| GSM4037886 | C1 |
| GSM4037888 | C1 |
| GSM4037889 | C1 |
| GSM4037890 | C1 |
| GSM4037891 | C1 |
| GSM4037892 | C1 |
| GSM4037893 | C1 |
| GSM4037894 | C1 |
| GSM4037895 | C1 |
| GSM4037896 | C1 |
| GSM4037901 | C1 |
| GSM4037913 | C1 |
| GSM4037925 | C1 |
| GSM4037933 | C1 |
| GSM4037939 | C1 |
| GSM1942590 | C1 |
| GSM1942591 | C1 |
| GSM1942592 | C1 |
| GSM1942593 | C1 |
| GSM1942594 | C1 |
| GSM1942595 | C1 |
| GSM1942598 | C1 |
| GSM1942612 | C1 |
| GSM1942613 | C1 |
| GSM1942614 | C1 |
| GSM1942616 | C1 |
| GSM1942617 | C1 |
| GSM1942620 | C1 |
| GSM4037882 | C2 |
| GSM4037885 | C2 |
| GSM4037887 | C2 |
| GSM4037897 | C2 |
| GSM4037899 | C2 |
| GSM4037903 | C2 |
| GSM4037905 | C2 |
| GSM4037907 | C2 |
| GSM4037909 | C2 |
| GSM4037911 | C2 |
| GSM4037915 | C2 |
| GSM4037917 | C2 |
| GSM4037919 | C2 |
| GSM4037921 | C2 |
| GSM4037923 | C2 |
| GSM4037927 | C2 |
| GSM4037929 | C2 |
| GSM4037931 | C2 |
| GSM4037935 | C2 |
| GSM4037937 | C2 |
| GSM4037941 | C2 |
| GSM4037943 | C2 |
| GSM1942596 | C2 |
| GSM1942597 | C2 |
| GSM1942599 | C2 |
| GSM1942603 | C2 |
| GSM1942604 | C2 |
| GSM1942605 | C2 |
| GSM1942606 | C2 |
| GSM1942609 | C2 |
| GSM1942610 | C2 |
| GSM1942611 | C2 |
| GSM1942618 | C2 |
| GSM1942619 | C2 |
| GSM1942621 | C2 |
| GSM1942622 | C2 |
| GSM1942623 | C2 |
| GSM1942624 | C2 |
| GSM1942626 | C2 |
| GSM1942627 | C2 |
| GSM1942632 | C2 |
| GSM1942633 | C2 |
| GSM1942637 | C2 |

Table S15. Results of DEGs clusters in Sarcoidosis

| ID | DEGs cluster |
| --- | --- |
| GSM2203587 | C1 |
| GSM2203588 | C1 |
| GSM2203589 | C1 |
| GSM2203590 | C1 |
| GSM2203591 | C1 |
| GSM2203592 | C1 |
| GSM2203593 | C1 |
| GSM2203594 | C1 |
| GSM2203595 | C1 |
| GSM2203596 | C1 |
| GSM2203597 | C1 |
| GSM2203598 | C1 |
| GSM2203599 | C1 |
| GSM2203600 | C1 |
| GSM2203602 | C1 |
| GSM2203603 | C1 |
| GSM2203604 | C1 |
| GSM2203605 | C1 |
| GSM2203609 | C1 |
| GSM2203610 | C1 |
| GSM2203611 | C1 |
| GSM2203612 | C1 |
| GSM2203613 | C1 |
| GSM2203614 | C1 |
| GSM2203616 | C1 |
| GSM2203619 | C1 |
| GSM2203620 | C1 |
| GSM2203621 | C1 |
| GSM2203622 | C1 |
| GSM2203623 | C1 |
| GSM2203624 | C1 |
| GSM2203625 | C1 |
| GSM2203626 | C1 |
| GSM2203627 | C1 |
| GSM2203628 | C1 |
| GSM2203629 | C1 |
| GSM2203630 | C1 |
| GSM2203631 | C1 |
| GSM2203632 | C1 |
| GSM2203633 | C1 |
| GSM2203634 | C1 |
| GSM2203635 | C1 |
| GSM2203636 | C1 |
| GSM2203637 | C1 |
| GSM2203638 | C1 |
| GSM2203639 | C1 |
| GSM2203640 | C1 |
| GSM2203641 | C1 |
| GSM2203642 | C1 |
| GSM2203643 | C1 |
| GSM2203644 | C1 |
| GSM2203645 | C1 |
| GSM2203646 | C1 |
| GSM2203647 | C1 |
| GSM2203695 | C1 |
| GSM2203696 | C1 |
| GSM2203698 | C1 |
| GSM2203711 | C1 |
| GSM2203718 | C1 |
| GSM2203733 | C1 |
| GSM2203736 | C1 |
| GSM2203737 | C1 |
| GSM2203738 | C1 |
| GSM2203739 | C1 |
| GSM2203740 | C1 |
| GSM479917 | C1 |
| GSM479920 | C1 |
| GSM479924 | C1 |
| GSM479926 | C1 |
| GSM479933 | C1 |
| GSM479935 | C1 |
| GSM479942 | C1 |
| GSM479944 | C1 |
| GSM479945 | C1 |
| GSM479949 | C1 |
| GSM479952 | C1 |
| GSM479953 | C1 |
| GSM479956 | C1 |
| GSM479957 | C1 |
| GSM479959 | C1 |
| GSM479961 | C1 |
| GSM479962 | C1 |
| GSM479963 | C1 |
| GSM479964 | C1 |
| GSM479965 | C1 |
| GSM479968 | C1 |
| GSM479969 | C1 |
| GSM479970 | C1 |
| GSM479971 | C1 |
| GSM479973 | C1 |
| GSM479974 | C1 |
| GSM479977 | C1 |
| GSM2203601 | C2 |
| GSM2203606 | C2 |
| GSM2203607 | C2 |
| GSM2203608 | C2 |
| GSM2203615 | C2 |
| GSM2203617 | C2 |
| GSM2203618 | C2 |
| GSM2203693 | C2 |
| GSM2203694 | C2 |
| GSM2203697 | C2 |
| GSM2203699 | C2 |
| GSM2203700 | C2 |
| GSM2203701 | C2 |
| GSM2203702 | C2 |
| GSM2203703 | C2 |
| GSM2203704 | C2 |
| GSM2203705 | C2 |
| GSM2203706 | C2 |
| GSM2203707 | C2 |
| GSM2203708 | C2 |
| GSM2203709 | C2 |
| GSM2203710 | C2 |
| GSM2203712 | C2 |
| GSM2203713 | C2 |
| GSM2203714 | C2 |
| GSM2203715 | C2 |
| GSM2203716 | C2 |
| GSM2203717 | C2 |
| GSM2203719 | C2 |
| GSM2203720 | C2 |
| GSM2203721 | C2 |
| GSM2203722 | C2 |
| GSM2203723 | C2 |
| GSM2203724 | C2 |
| GSM2203725 | C2 |
| GSM2203726 | C2 |
| GSM2203727 | C2 |
| GSM2203728 | C2 |
| GSM2203729 | C2 |
| GSM2203730 | C2 |
| GSM2203731 | C2 |
| GSM2203732 | C2 |
| GSM2203734 | C2 |
| GSM2203735 | C2 |
| GSM2203741 | C2 |
| GSM479919 | C2 |
| GSM479921 | C2 |
| GSM479922 | C2 |
| GSM479923 | C2 |
| GSM479925 | C2 |
| GSM479927 | C2 |
| GSM479928 | C2 |
| GSM479931 | C2 |
| GSM479932 | C2 |
| GSM479934 | C2 |
| GSM479936 | C2 |
| GSM479937 | C2 |
| GSM479939 | C2 |
| GSM479940 | C2 |
| GSM479941 | C2 |
| GSM479943 | C2 |
| GSM479946 | C2 |
| GSM479947 | C2 |
| GSM479948 | C2 |
| GSM479951 | C2 |
| GSM479954 | C2 |
| GSM479958 | C2 |
| GSM479960 | C2 |
| GSM479966 | C2 |
| GSM479967 | C2 |
| GSM479972 | C2 |
| GSM479975 | C2 |
| GSM479976 | C2 |
| GSM479979 | C2 |
| GSM479980 | C2 |
| GSM479981 | C2 |

Table S16. Results of differential expression analysis between DEGs clusters in sarcoidosis

|  | logFC | AveExpr | t | P.Value | adj.P.Val | B |
| --- | --- | --- | --- | --- | --- | --- |
| LTF | 2.590949 | 2.322965 | 15.81883 | 2.84E-35 | 4.6E-31 | 68.60347 |
| CEACAM8 | 2.686492 | 1.99531 | 15.55721 | 1.53E-34 | 1.24E-30 | 66.98687 |
| ELANE | 2.507761 | 1.563313 | 14.13226 | 1.62E-30 | 8.76E-27 | 58.08546 |
| CEACAM6 | 2.507471 | 1.762057 | 13.93746 | 5.8E-30 | 2.35E-26 | 56.85838 |
| OLR1 | 2.146353 | 1.513619 | 13.56392 | 6.71E-29 | 2.17E-25 | 54.50048 |
| CAMP | 1.948026 | 2.047865 | 13.15165 | 1E-27 | 2.71E-24 | 51.8924 |
| CTSG | 2.540622 | 2.312371 | 12.79575 | 1.04E-26 | 2.41E-23 | 49.63802 |
| DEFA4 | 2.564994 | 2.174475 | 12.63669 | 2.95E-26 | 5.98E-23 | 48.63014 |
| COL17A1 | 1.765673 | 1.846161 | 12.1244 | 8.51E-25 | 1.53E-21 | 45.3847 |
| MPO | 2.112562 | 1.919208 | 11.93314 | 2.98E-24 | 4.83E-21 | 44.17419 |
| LCN2 | 1.658424 | 2.052068 | 11.41894 | 8.58E-23 | 1.26E-19 | 40.92664 |
| RNASE3 | 2.36384 | 2.086645 | 11.39684 | 9.9E-23 | 1.34E-19 | 40.78737 |
| TCN1 | 1.636883 | 1.812258 | 10.87574 | 2.93E-21 | 3.65E-18 | 37.51293 |
| AZU1 | 1.740149 | 1.926178 | 9.466678 | 2.36E-17 | 2.73E-14 | 28.80753 |
| MMP8 | 1.346625 | 1.485483 | 9.023293 | 3.73E-16 | 4.04E-13 | 26.13552 |
| RETN | 1.799837 | 2.297268 | 8.470051 | 1.1E-14 | 1.05E-11 | 22.86385 |
| PGLYRP1 | 1.341506 | 1.936981 | 8.04116 | 1.43E-13 | 9.65E-11 | 20.38385 |
| RNASE2 | 1.333242 | 2.200821 | 7.664217 | 1.3E-12 | 5.01E-10 | 18.25115 |
| MPL | 1.182488 | 2.0178 | 7.560038 | 2.37E-12 | 8.01E-10 | 17.67019 |
| IGF2BP3 | 1.10611 | 0.909494 | 7.46146 | 4.18E-12 | 1.07E-09 | 17.12403 |
| HP | 1.469408 | 1.783776 | 7.442469 | 4.65E-12 | 1.13E-09 | 17.01922 |
| PROS1 | 1.296633 | 1.770699 | 7.424143 | 5.17E-12 | 1.23E-09 | 16.9182 |
| GP6 | 1.285997 | 1.870007 | 7.414412 | 5.46E-12 | 1.24E-09 | 16.86462 |
| PRTN3 | 1.043339 | 1.520385 | 7.389895 | 6.28E-12 | 1.34E-09 | 16.72976 |
| CSF2RB | 1.110996 | 2.714606 | 7.364654 | 7.25E-12 | 1.43E-09 | 16.59115 |
| PDK4 | 1.203312 | 1.855144 | 7.363824 | 7.29E-12 | 1.43E-09 | 16.5866 |
| JAM3 | 1.169546 | 2.022261 | 7.249749 | 1.39E-11 | 2.23E-09 | 15.96325 |
| SOCS1 | 1.109103 | 1.458734 | 7.241896 | 1.45E-11 | 2.25E-09 | 15.92052 |
| C1QB | 1.660972 | 1.128501 | 7.215828 | 1.68E-11 | 2.53E-09 | 15.77886 |
| LPCAT2 | 1.289196 | 1.915539 | 7.154003 | 2.38E-11 | 3.11E-09 | 15.44395 |
| GBP6 | 1.605187 | 0.847784 | 7.027119 | 4.84E-11 | 4.93E-09 | 14.76141 |
| ARRDC3 | 1.320113 | 2.373275 | 7.018224 | 5.08E-11 | 5.08E-09 | 14.71381 |
| CDR2L | 1.135459 | 1.834086 | 7.015616 | 5.15E-11 | 5.08E-09 | 14.69985 |
| GPR84 | 1.028142 | 1.340683 | 7.005264 | 5.46E-11 | 5.24E-09 | 14.64451 |
| LHFPL2 | 1.104495 | 1.650933 | 7.004004 | 5.5E-11 | 5.24E-09 | 14.63777 |
| SAMD14 | 1.219566 | 1.464902 | 6.994591 | 5.79E-11 | 5.43E-09 | 14.58749 |
| TREML1 | 1.06239 | 2.057984 | 6.985963 | 6.08E-11 | 5.59E-09 | 14.54143 |
| SH2D3C | 1.074395 | 1.93347 | 6.968383 | 6.7E-11 | 5.93E-09 | 14.44768 |
| TAP2 | 1.095825 | 1.626088 | 6.95202 | 7.33E-11 | 6.33E-09 | 14.36054 |
| CEACAM1 | 1.1101 | 1.684133 | 6.938225 | 7.91E-11 | 6.62E-09 | 14.28716 |
| ANKRD22 | 1.756248 | 1.243204 | 6.937076 | 7.96E-11 | 6.62E-09 | 14.28106 |
| LILRB1 | 1.107398 | 2.12412 | 6.925199 | 8.5E-11 | 6.92E-09 | 14.21794 |
| BATF2 | 1.54542 | 1.167363 | 6.924218 | 8.55E-11 | 6.92E-09 | 14.21274 |
| UBE2H | 1.042866 | 2.347775 | 6.908895 | 9.3E-11 | 7.39E-09 | 14.13141 |
| DTX3L | 1.315796 | 2.103214 | 6.864941 | 1.18E-10 | 8.69E-09 | 13.89869 |
| PLOD2 | 1.030853 | 1.274568 | 6.838182 | 1.37E-10 | 9.62E-09 | 13.75742 |
| GPR27 | 1.004716 | 1.580566 | 6.793544 | 1.75E-10 | 1.15E-08 | 13.52244 |
| GYPB | 1.467215 | 2.407225 | 6.783581 | 1.85E-10 | 1.19E-08 | 13.47011 |
| FBXO6 | 1.075487 | 1.313713 | 6.780228 | 1.88E-10 | 1.19E-08 | 13.45251 |
| MARCO | 1.096851 | 1.788854 | 6.751077 | 2.21E-10 | 1.32E-08 | 13.2997 |
| SLC6A9 | 1.332822 | 2.228086 | 6.725446 | 2.53E-10 | 1.47E-08 | 13.16565 |
| VWF | 1.1139 | 1.757794 | 6.712701 | 2.72E-10 | 1.56E-08 | 13.0991 |
| CDKN2D | 1.043089 | 2.047491 | 6.676441 | 3.31E-10 | 1.8E-08 | 12.91016 |
| TRIM58 | 1.236779 | 2.262099 | 6.648021 | 3.85E-10 | 1.98E-08 | 12.76248 |
| IFIT3 | 1.464063 | 1.727033 | 6.63059 | 4.23E-10 | 2.13E-08 | 12.67209 |
| SCO2 | 1.029494 | 1.612885 | 6.630055 | 4.24E-10 | 2.13E-08 | 12.66932 |
| FANCL | 1.032953 | 1.951884 | 6.595154 | 5.12E-10 | 2.46E-08 | 12.48875 |
| HES4 | 1.136234 | 1.940512 | 6.591332 | 5.23E-10 | 2.49E-08 | 12.46901 |
| CEBPE | 1.219335 | 2.139857 | 6.584409 | 5.43E-10 | 2.57E-08 | 12.43327 |
| SERPING1 | 1.601196 | 1.161753 | 6.566322 | 5.98E-10 | 2.79E-08 | 12.34001 |
| ITGB3 | 1.159295 | 1.75153 | 6.565696 | 6E-10 | 2.79E-08 | 12.33678 |
| CTNNAL1 | 1.20517 | 2.059463 | 6.540297 | 6.87E-10 | 3.1E-08 | 12.20607 |
| TCN2 | 1.067515 | 1.561042 | 6.537356 | 6.98E-10 | 3.12E-08 | 12.19095 |
| PLSCR1 | 1.096674 | 1.830792 | 6.522839 | 7.54E-10 | 3.29E-08 | 12.1164 |
| VMO1 | 1.007676 | 1.827132 | 6.516189 | 7.81E-10 | 3.37E-08 | 12.08228 |
| APOL6 | 1.223226 | 2.434663 | 6.494242 | 8.78E-10 | 3.68E-08 | 11.96982 |
| GRAMD1B | 1.179148 | 1.603047 | 6.48969 | 9E-10 | 3.72E-08 | 11.94652 |
| DDX60 | 1.079662 | 1.783558 | 6.487241 | 9.12E-10 | 3.74E-08 | 11.93399 |
| C2 | 1.048901 | 1.160964 | 6.486956 | 9.13E-10 | 3.74E-08 | 11.93254 |
| ETV7 | 1.114676 | 1.340966 | 6.424795 | 1.27E-09 | 4.73E-08 | 11.61546 |
| SLC2A5 | 1.012487 | 1.64489 | 6.39423 | 1.49E-09 | 5.34E-08 | 11.46023 |
| CXCL10 | 1.413483 | 1.485244 | 6.365947 | 1.73E-09 | 5.96E-08 | 11.31698 |
| AIM2 | 1.083804 | 1.837827 | 6.359466 | 1.79E-09 | 6.08E-08 | 11.28421 |
| GBP4 | 1.135914 | 1.805465 | 6.334029 | 2.05E-09 | 6.67E-08 | 11.15579 |
| TMEM56 | 1.392338 | 1.781479 | 6.319274 | 2.21E-09 | 7.03E-08 | 11.08144 |
| KREMEN1 | 1.149926 | 1.670502 | 6.305522 | 2.38E-09 | 7.47E-08 | 11.01224 |
| WARS | 1.102163 | 1.870608 | 6.29875 | 2.46E-09 | 7.67E-08 | 10.9782 |
| IDO1 | 1.34286 | 1.5951 | 6.292739 | 2.54E-09 | 7.79E-08 | 10.948 |
| FCGR1A | 1.301737 | 1.396391 | 6.265512 | 2.93E-09 | 8.62E-08 | 10.81144 |
| SUCNR1 | 1.031845 | 1.473841 | 6.263397 | 2.96E-09 | 8.67E-08 | 10.80085 |
| NDUFAF3 | 1.022756 | 1.604902 | 6.243949 | 3.28E-09 | 9.28E-08 | 10.70355 |
| MX1 | 1.037579 | 1.856134 | 6.168187 | 4.86E-09 | 1.27E-07 | 10.3263 |
| RTP4 | 1.17388 | 1.647691 | 6.156163 | 5.17E-09 | 1.33E-07 | 10.26669 |
| OASL | 1.0086 | 1.732294 | 6.106927 | 6.66E-09 | 1.59E-07 | 10.02338 |
| TIMM10 | 1.240037 | 2.100584 | 6.092514 | 7.18E-09 | 1.7E-07 | 9.952388 |
| SLC7A5 | 1.210356 | 2.140571 | 6.079665 | 7.66E-09 | 1.78E-07 | 9.889185 |
| ISG15 | 1.199714 | 1.759551 | 6.0773 | 7.76E-09 | 1.8E-07 | 9.877564 |
| BCAT1 | 1.012432 | 1.815024 | 6.073304 | 7.92E-09 | 1.83E-07 | 9.857931 |
| IFI27 | 1.680164 | 1.914944 | 6.065905 | 8.22E-09 | 1.89E-07 | 9.8216 |
| HERC5 | 1.036188 | 1.751297 | 6.04269 | 9.26E-09 | 2.07E-07 | 9.707797 |
| SLC22A16 | 1.025357 | 1.625894 | 6.013526 | 1.07E-08 | 2.32E-07 | 9.565225 |
| EPSTI1 | 1.098345 | 1.565303 | 6.0001 | 1.15E-08 | 2.43E-07 | 9.49974 |
| RAB20 | 1.060129 | 1.693914 | 5.986973 | 1.23E-08 | 2.57E-07 | 9.435802 |
| FCGR1B | 1.263595 | 1.736648 | 5.980366 | 1.27E-08 | 2.64E-07 | 9.403655 |
| ITGA2B | 1.055958 | 2.064487 | 5.963738 | 1.38E-08 | 2.82E-07 | 9.322855 |
| GYPE | 1.192814 | 1.515471 | 5.956638 | 1.43E-08 | 2.89E-07 | 9.288399 |
| SPOCD1 | 1.007078 | 1.275574 | 5.943597 | 1.53E-08 | 3.04E-07 | 9.225179 |
| DHRS9 | 1.145363 | 1.977552 | 5.885221 | 2.06E-08 | 3.87E-07 | 8.943294 |
| IFITM3 | 1.065568 | 2.09681 | 5.800521 | 3.14E-08 | 5.35E-07 | 8.537545 |
| ARL4A | 1.0697 | 2.525941 | 5.756499 | 3.91E-08 | 6.35E-07 | 8.328204 |
| RPL3L | 1.196646 | 1.80051 | 5.754626 | 3.94E-08 | 6.39E-07 | 8.31932 |
| IFI44L | 1.337238 | 1.640831 | 5.744194 | 4.15E-08 | 6.61E-07 | 8.269878 |
| GBP5 | 1.118863 | 2.144618 | 5.743324 | 4.17E-08 | 6.63E-07 | 8.265756 |
| OAS3 | 1.159634 | 1.83174 | 5.666369 | 6.1E-08 | 8.98E-07 | 7.90293 |
| GBP1 | 1.097711 | 1.975633 | 5.660998 | 6.26E-08 | 9.16E-07 | 7.87773 |
| TMCC2 | 1.16871 | 1.974013 | 5.659224 | 6.31E-08 | 9.21E-07 | 7.86941 |
| SLAMF8 | 1.003948 | 1.498229 | 5.531047 | 1.18E-07 | 1.55E-06 | 7.272998 |
| RAP1GAP | 1.797631 | 2.126223 | 5.516883 | 1.26E-07 | 1.64E-06 | 7.207667 |
| RNF175 | 1.313861 | 2.548835 | 5.50936 | 1.31E-07 | 1.68E-06 | 7.173014 |
| YOD1 | 1.151619 | 2.224897 | 5.491747 | 1.42E-07 | 1.8E-06 | 7.092014 |
| TOR2A | 1.163241 | 2.427414 | 5.455612 | 1.69E-07 | 2.08E-06 | 6.926396 |
| ARG1 | 1.206963 | 2.152922 | 5.446001 | 1.77E-07 | 2.17E-06 | 6.882475 |
| CMPK2 | 1.09954 | 2.128738 | 5.441685 | 1.81E-07 | 2.2E-06 | 6.862766 |
| ZDHHC19 | 1.003963 | 1.814103 | 5.428001 | 1.93E-07 | 2.32E-06 | 6.800361 |
| IFI44 | 1.007377 | 1.492269 | 5.4246 | 1.96E-07 | 2.34E-06 | 6.784867 |
| SLC4A1 | 1.05856 | 2.452047 | 5.406696 | 2.14E-07 | 2.52E-06 | 6.703416 |
| IFIT1 | 1.083928 | 2.170081 | 5.390351 | 2.31E-07 | 2.69E-06 | 6.629222 |
| KLC3 | 1.045845 | 2.152412 | 5.379662 | 2.43E-07 | 2.81E-06 | 6.580784 |
| ARHGEF12 | 1.092258 | 1.507628 | 5.365608 | 2.6E-07 | 2.98E-06 | 6.517204 |
| ALAS2 | 1.250612 | 2.947662 | 5.338867 | 2.95E-07 | 3.29E-06 | 6.396555 |
| RSAD2 | 1.233406 | 1.774539 | 5.336849 | 2.98E-07 | 3.32E-06 | 6.387469 |
| OTOF | 1.004034 | 1.491482 | 5.279088 | 3.91E-07 | 4.22E-06 | 6.12839 |
| OAS1 | 1.065526 | 1.944986 | 5.273277 | 4.02E-07 | 4.32E-06 | 6.102435 |
| CCRN4L | 1.104805 | 1.948515 | 5.194711 | 5.8E-07 | 5.94E-06 | 5.753553 |
| KANK2 | 1.013915 | 1.937333 | 5.156315 | 6.92E-07 | 6.94E-06 | 5.584423 |
| CYP4F22 | 1.026953 | 2.244287 | 5.010588 | 1.35E-06 | 1.21E-05 | 4.950851 |
| ANK1 | 1.044 | 2.200549 | 4.998792 | 1.42E-06 | 1.27E-05 | 4.900153 |
| UGP2 | 1.205644 | 2.651062 | 4.989377 | 1.48E-06 | 1.32E-05 | 4.859747 |
| RHAG | 1.005129 | 1.659973 | 4.971082 | 1.61E-06 | 1.42E-05 | 4.781399 |
| THBS1 | 1.008236 | 1.942359 | 4.951664 | 1.76E-06 | 1.53E-05 | 4.69847 |
| CHTF8 | 1.259591 | 2.562854 | 4.895688 | 2.26E-06 | 1.9E-05 | 4.460775 |
| TPST1 | 1.069396 | 2.061432 | 4.850023 | 2.77E-06 | 2.25E-05 | 4.268361 |
| NFIX | 1.11966 | 2.524152 | 4.830375 | 3.02E-06 | 2.43E-05 | 4.185993 |
| HLA-DRB6 | 1.320401 | 1.752048 | 4.743969 | 4.41E-06 | 3.35E-05 | 3.826759 |
| CCL23 | 1.122715 | 1.816855 | 4.492276 | 1.3E-05 | 8.45E-05 | 2.80882 |
| PF4V1 | 1.107492 | 1.737146 | 4.464387 | 1.46E-05 | 9.36E-05 | 2.698685 |
| CASC1 | 1.088102 | 2.182106 | 4.143838 | 5.37E-05 | 0.000288 | 1.472208 |
| RNF182 | 1.32988 | 2.13412 | 3.712834 | 0.000277 | 0.001197 | -0.0583 |
| CMBL | 1.011515 | 1.960647 | 3.632084 | 0.000372 | 0.001549 | -0.32932 |
| RPA4 | -1.20107 | 1.713272 | -8.62174 | 4.38E-15 | 4.44E-12 | 23.75327 |
| NT5E | -1.58519 | 1.851866 | -8.4134 | 1.55E-14 | 1.39E-11 | 22.53328 |
| KLF12 | -1.07794 | 2.291564 | -8.3727 | 1.98E-14 | 1.69E-11 | 22.29631 |
| CD40LG | -1.25215 | 2.140825 | -8.31778 | 2.75E-14 | 2.23E-11 | 21.9773 |
| ZNF239 | -1.19774 | 1.920479 | -8.24044 | 4.37E-14 | 3.37E-11 | 21.52951 |
| CRYGS | -1.06064 | 1.976695 | -8.11578 | 9.18E-14 | 6.76E-11 | 20.81141 |
| ZNF827 | -1.13259 | 2.022928 | -8.0306 | 1.52E-13 | 9.86E-11 | 20.3235 |
| TSPYL2 | -1.31449 | 2.170327 | -7.94524 | 2.52E-13 | 1.46E-10 | 19.83674 |
| ZNF550 | -1.01697 | 2.163901 | -7.93037 | 2.75E-13 | 1.54E-10 | 19.75223 |
| ZNF91 | -1.39481 | 2.126028 | -7.83243 | 4.88E-13 | 2.47E-10 | 19.19711 |
| RPS18 | -1.02346 | 3.104088 | -7.78946 | 6.27E-13 | 2.99E-10 | 18.95453 |
| FAM171A1 | -1.04714 | 1.936582 | -7.78293 | 6.51E-13 | 3.02E-10 | 18.91775 |
| SLC41A1 | -1.17717 | 2.103336 | -7.69348 | 1.1E-12 | 4.45E-10 | 18.415 |
| TRIB2 | -1.03155 | 2.585025 | -7.693 | 1.1E-12 | 4.45E-10 | 18.41236 |
| LOC100131662 | -1.39835 | 2.061711 | -7.62947 | 1.59E-12 | 5.87E-10 | 18.05695 |
| COCH | -1.51733 | 2.952421 | -7.62508 | 1.63E-12 | 5.87E-10 | 18.03247 |
| ZFP82 | -1.12822 | 2.024792 | -7.61998 | 1.68E-12 | 5.91E-10 | 18.00402 |
| C12orf57 | -1.07732 | 2.363766 | -7.55168 | 2.49E-12 | 8.23E-10 | 17.62375 |
| KLHL3 | -1.08686 | 2.258768 | -7.53138 | 2.8E-12 | 8.71E-10 | 17.51106 |
| TPM2 | -1.11979 | 2.329724 | -7.52533 | 2.89E-12 | 8.85E-10 | 17.47749 |
| TLE2 | -1.20957 | 2.106234 | -7.50458 | 3.26E-12 | 9.27E-10 | 17.36248 |
| ZNF573 | -1.18525 | 1.934048 | -7.50107 | 3.33E-12 | 9.3E-10 | 17.34304 |
| SELM | -1.24623 | 1.912387 | -7.47257 | 3.92E-12 | 1.04E-09 | 17.18538 |
| ZNF831 | -1.03505 | 2.310064 | -7.4626 | 4.15E-12 | 1.07E-09 | 17.1303 |
| SLC16A10 | -1.49046 | 2.120576 | -7.41831 | 5.34E-12 | 1.24E-09 | 16.88607 |
| FAM35A | -1.16996 | 2.543535 | -7.41026 | 5.59E-12 | 1.24E-09 | 16.84175 |
| CD27 | -1.11913 | 2.52787 | -7.37327 | 6.91E-12 | 1.4E-09 | 16.63842 |
| ZNF540 | -1.21651 | 2.452273 | -7.34389 | 8.16E-12 | 1.56E-09 | 16.47731 |
| RLTPR | -1.13509 | 2.097443 | -7.33831 | 8.42E-12 | 1.59E-09 | 16.44678 |
| DOCK9 | -1.17494 | 2.232429 | -7.28197 | 1.16E-11 | 1.96E-09 | 16.13882 |
| INADL | -1.1913 | 2.475208 | -7.26145 | 1.3E-11 | 2.13E-09 | 16.02697 |
| PLEKHB1 | -1.00775 | 2.094575 | -7.2397 | 1.47E-11 | 2.25E-09 | 15.90856 |
| EPHX2 | -1.22052 | 2.135757 | -7.23936 | 1.47E-11 | 2.25E-09 | 15.90673 |
| SMYD2 | -1.15239 | 2.187943 | -7.21029 | 1.74E-11 | 2.57E-09 | 15.74881 |
| MDN1 | -1.05382 | 1.716651 | -7.20641 | 1.77E-11 | 2.57E-09 | 15.72774 |
| SLC7A6 | -1.04417 | 2.494212 | -7.1637 | 2.26E-11 | 3.02E-09 | 15.49638 |
| EDAR | -1.30436 | 2.102033 | -7.1325 | 2.69E-11 | 3.38E-09 | 15.32782 |
| CNTNAP1 | -1.10997 | 1.96514 | -7.09998 | 3.22E-11 | 3.81E-09 | 15.15255 |
| TCF7 | -1.36701 | 2.674226 | -7.08227 | 3.56E-11 | 4.04E-09 | 15.05725 |
| ZC3H12B | -1.10217 | 1.82569 | -7.01682 | 5.12E-11 | 5.08E-09 | 14.7063 |
| OSBPL10 | -1.38526 | 2.33594 | -6.99252 | 5.86E-11 | 5.45E-09 | 14.57641 |
| GPR183 | -1.1874 | 2.430975 | -6.98179 | 6.22E-11 | 5.69E-09 | 14.51916 |
| FAM159A | -1.00541 | 2.234905 | -6.97477 | 6.46E-11 | 5.82E-09 | 14.48173 |
| FAM102A | -1.1386 | 2.295265 | -6.94488 | 7.62E-11 | 6.44E-09 | 14.32254 |
| TBC1D4 | -1.04433 | 2.670452 | -6.90553 | 9.47E-11 | 7.49E-09 | 14.11359 |
| C14orf132 | -1.22075 | 2.071452 | -6.8864 | 1.05E-10 | 8.16E-09 | 14.01223 |
| CAMK2N1 | -1.04715 | 2.378147 | -6.82098 | 1.51E-10 | 1.02E-08 | 13.66677 |
| TMEM204 | -1.03841 | 2.431756 | -6.81504 | 1.56E-10 | 1.04E-08 | 13.63551 |
| ID3 | -1.05632 | 2.258415 | -6.769 | 2E-10 | 1.24E-08 | 13.39361 |
| IL23A | -1.096 | 2.340924 | -6.7441 | 2.29E-10 | 1.35E-08 | 13.26316 |
| RPL14 | -1.01527 | 3.140779 | -6.70456 | 2.84E-10 | 1.61E-08 | 13.05663 |
| ZNF420 | -1.1317 | 2.080027 | -6.67246 | 3.38E-10 | 1.81E-08 | 12.88947 |
| GZMK | -1.05156 | 2.438273 | -6.65948 | 3.62E-10 | 1.91E-08 | 12.82196 |
| DTX3 | -1.06027 | 2.078409 | -6.65544 | 3.7E-10 | 1.94E-08 | 12.801 |
| C5orf28 | -1.12332 | 2.127825 | -6.65063 | 3.8E-10 | 1.96E-08 | 12.77601 |
| SLC39A10 | -1.00315 | 2.254239 | -6.64527 | 3.91E-10 | 2E-08 | 12.74819 |
| MKL2 | -1.03843 | 1.958712 | -6.62952 | 4.26E-10 | 2.13E-08 | 12.66654 |
| C11orf80 | -1.01155 | 2.082692 | -6.62402 | 4.38E-10 | 2.17E-08 | 12.63807 |
| HOOK1 | -1.15411 | 2.31302 | -6.62005 | 4.48E-10 | 2.21E-08 | 12.6175 |
| PLEKHG4 | -1.05266 | 2.082995 | -6.56235 | 6.11E-10 | 2.83E-08 | 12.31952 |
| LRRN3 | -1.87905 | 2.267616 | -6.55789 | 6.25E-10 | 2.88E-08 | 12.29656 |
| FAM60A | -1.10468 | 2.448633 | -6.53615 | 7.02E-10 | 3.13E-08 | 12.18475 |
| CD248 | -1.32927 | 2.01998 | -6.5295 | 7.28E-10 | 3.2E-08 | 12.1506 |
| CBR3 | -1.08201 | 2.245037 | -6.51842 | 7.72E-10 | 3.35E-08 | 12.09371 |
| ALDOB | -1.05384 | 1.677327 | -6.49766 | 8.63E-10 | 3.65E-08 | 11.98735 |
| APBA2 | -1.00688 | 2.30806 | -6.46791 | 1.01E-09 | 3.98E-08 | 11.83517 |
| LEF1 | -1.05903 | 2.468824 | -6.46194 | 1.04E-09 | 4.08E-08 | 11.8047 |
| ZFP42 | -1.10115 | 1.888154 | -6.45662 | 1.07E-09 | 4.18E-08 | 11.77755 |
| FBLN5 | -1.13571 | 2.312947 | -6.44996 | 1.11E-09 | 4.28E-08 | 11.74361 |
| MGC40069 | -1.07085 | 1.992773 | -6.44686 | 1.13E-09 | 4.34E-08 | 11.72782 |
| EPHA1 | -1.12252 | 2.101443 | -6.42035 | 1.3E-09 | 4.76E-08 | 11.59285 |
| KRT73 | -1.32313 | 2.062258 | -6.36636 | 1.73E-09 | 5.96E-08 | 11.31905 |
| CAMTA1 | -1.07726 | 1.996114 | -6.36451 | 1.75E-09 | 5.98E-08 | 11.3097 |
| CAMK4 | -1.01847 | 2.324097 | -6.30009 | 2.45E-09 | 7.67E-08 | 10.98491 |
| CLSTN3 | -1.16161 | 2.128083 | -6.29577 | 2.5E-09 | 7.71E-08 | 10.96324 |
| NCR3 | -1.00492 | 2.483229 | -6.25042 | 3.17E-09 | 9.12E-08 | 10.73592 |
| AKAP7 | -1.05928 | 2.251433 | -6.24749 | 3.22E-09 | 9.17E-08 | 10.72127 |
| TCEA3 | -1.14031 | 2.300928 | -6.19762 | 4.17E-09 | 1.13E-07 | 10.47252 |
| LAMA5 | -1.04713 | 2.187463 | -6.17922 | 4.59E-09 | 1.23E-07 | 10.38104 |
| TRIM32 | -1.00653 | 2.03354 | -6.17511 | 4.69E-09 | 1.24E-07 | 10.36065 |
| AXIN2 | -1.00064 | 2.186936 | -6.15204 | 5.28E-09 | 1.34E-07 | 10.24628 |
| CCR7 | -1.03283 | 2.495735 | -6.14952 | 5.35E-09 | 1.36E-07 | 10.23381 |
| CEP68 | -1.06172 | 2.42604 | -6.13223 | 5.85E-09 | 1.45E-07 | 10.14825 |
| PARM1 | -1.06425 | 2.197548 | -6.06372 | 8.32E-09 | 1.9E-07 | 9.81087 |
| IGSF9B | -1.01577 | 1.756194 | -6.05996 | 8.48E-09 | 1.93E-07 | 9.792424 |
| FHIT | -1.132 | 2.391383 | -6.01478 | 1.07E-08 | 2.3E-07 | 9.571347 |
| AHI1 | -1.06914 | 2.119113 | -5.96349 | 1.39E-08 | 2.82E-07 | 9.321668 |
| RAPGEF1 | -1.08262 | 2.226615 | -5.93862 | 1.57E-08 | 3.1E-07 | 9.201053 |
| PURA | -1.02411 | 2.39574 | -5.93122 | 1.63E-08 | 3.19E-07 | 9.165268 |
| FAM3C | -1.0303 | 2.395867 | -5.88846 | 2.02E-08 | 3.83E-07 | 8.958863 |
| DSC1 | -1.1848 | 2.072644 | -5.81395 | 2.94E-08 | 5.1E-07 | 8.6016 |
| TSHZ2 | -1.17288 | 2.345677 | -5.7774 | 3.52E-08 | 5.8E-07 | 8.427472 |
| BLK | -1.01474 | 2.019167 | -5.71489 | 4.8E-08 | 7.42E-07 | 8.13133 |
| MGC24103 | -1.1366 | 2.048908 | -5.64826 | 6.66E-08 | 9.63E-07 | 7.818023 |
| RTKN2 | -1.00826 | 1.968727 | -5.63719 | 7.03E-08 | 1.01E-06 | 7.766218 |
| CRIP2 | -1.32219 | 2.418234 | -5.61283 | 7.92E-08 | 1.11E-06 | 7.652482 |
| C22orf34 | -1.04819 | 2.048607 | -5.61053 | 8.01E-08 | 1.13E-06 | 7.641739 |
| CCDC15 | -1.03148 | 2.298367 | -5.56436 | 1E-07 | 1.36E-06 | 7.42711 |
| HMGB1 | -1.2752 | 2.778247 | -5.50028 | 1.37E-07 | 1.74E-06 | 7.131235 |
| HPCAL4 | -1.01463 | 1.906582 | -5.42791 | 1.93E-07 | 2.32E-06 | 6.79993 |
| KRT72 | -1.33774 | 2.235762 | -5.39881 | 2.22E-07 | 2.6E-06 | 6.667592 |
| CCDC58 | -1.05225 | 2.500516 | -5.18315 | 6.12E-07 | 6.21E-06 | 5.702537 |
| PTMA | -1.0731 | 2.984408 | -5.17425 | 6.37E-07 | 6.44E-06 | 5.663317 |
| RPLP1 | -1.02144 | 3.031571 | -5.14891 | 7.16E-07 | 7.12E-06 | 5.551894 |
| CLECL1 | -1.06203 | 2.419607 | -4.95299 | 1.75E-06 | 1.53E-05 | 4.704123 |
| ITGB1BP1 | -1.00401 | 2.34366 | -4.94979 | 1.77E-06 | 1.54E-05 | 4.690477 |
| CACYBP | -1.15909 | 2.775022 | -4.89384 | 2.28E-06 | 1.92E-05 | 4.452974 |
| SLC7A3 | -1.04708 | 1.801249 | -4.61034 | 7.85E-06 | 5.5E-05 | 3.280952 |
| CNTNAP2 | -1.06738 | 2.110578 | -3.96446 | 0.000108 | 0.000531 | 0.818303 |
| HBE1 | -1.09039 | 2.123106 | -3.29667 | 0.00119 | 0.004263 | -1.39993 |

Table S17. Results of differential expression analysis between DEGs clusters in OSA

|  | logFC | AveExpr | t | P.Value | adj.P.Val | B |
| --- | --- | --- | --- | --- | --- | --- |
| PMP2 | 1.074759 | 3.364828 | 4.333366 | 4.44E-05 | 0.238563 | 1.378592 |
| NUMBL | 1.004455 | 6.908642 | 3.636974 | 0.0005 | 0.397332 | -0.45939 |
| ZC3H12D | 1.011832 | 5.768847 | 3.359823 | 0.001222 | 0.397332 | -1.13429 |
| PPL | 1.030991 | 6.132619 | 2.88235 | 0.00513 | 0.439453 | -2.20747 |
| NDUFA5 | -1.13188 | 5.831837 | -3.94398 | 0.000177 | 0.238563 | 0.327691 |
| IDO1 | -1.22117 | 5.453554 | -3.50062 | 0.00078 | 0.397332 | -0.79589 |
| RD3 | -1.02024 | 4.786992 | -3.48465 | 0.000821 | 0.397332 | -0.83475 |
| RBAK | -1.06086 | 5.955061 | -3.33662 | 0.001315 | 0.397332 | -1.18915 |
| AADAC | -1.03882 | 4.43986 | -2.70176 | 0.008502 | 0.472191 | -2.58089 |
| MMP8 | -1.07375 | 3.639064 | -2.38269 | 0.019687 | 0.539756 | -3.1931 |

Table S18. Results of DEAG clusters in OSA

| ID | DEAG cluster |
| --- | --- |
| GSM4037879 | CI |
| GSM4037880 | CI |
| GSM4037885 | CI |
| GSM4037888 | CI |
| GSM4037889 | CI |
| GSM4037890 | CI |
| GSM4037894 | CI |
| GSM4037903 | CI |
| GSM4037905 | CI |
| GSM4037907 | CI |
| GSM4037909 | CI |
| GSM4037911 | CI |
| GSM4037915 | CI |
| GSM4037917 | CI |
| GSM4037919 | CI |
| GSM4037921 | CI |
| GSM4037925 | CI |
| GSM4037927 | CI |
| GSM4037931 | CI |
| GSM4037935 | CI |
| GSM1942596 | CI |
| GSM1942597 | CI |
| GSM1942599 | CI |
| GSM1942603 | CI |
| GSM1942604 | CI |
| GSM1942605 | CI |
| GSM1942606 | CI |
| GSM1942609 | CI |
| GSM1942611 | CI |
| GSM1942619 | CI |
| GSM1942621 | CI |
| GSM1942622 | CI |
| GSM1942623 | CI |
| GSM1942626 | CI |
| GSM1942627 | CI |
| GSM1942637 | CI |
| GSM4037881 | CII |
| GSM4037882 | CII |
| GSM4037883 | CII |
| GSM4037884 | CII |
| GSM4037886 | CII |
| GSM4037887 | CII |
| GSM4037891 | CII |
| GSM4037892 | CII |
| GSM4037893 | CII |
| GSM4037895 | CII |
| GSM4037896 | CII |
| GSM4037897 | CII |
| GSM4037899 | CII |
| GSM4037901 | CII |
| GSM4037913 | CII |
| GSM4037923 | CII |
| GSM4037929 | CII |
| GSM4037933 | CII |
| GSM4037937 | CII |
| GSM4037939 | CII |
| GSM4037941 | CII |
| GSM4037943 | CII |
| GSM1942590 | CII |
| GSM1942591 | CII |
| GSM1942592 | CII |
| GSM1942593 | CII |
| GSM1942594 | CII |
| GSM1942595 | CII |
| GSM1942598 | CII |
| GSM1942610 | CII |
| GSM1942612 | CII |
| GSM1942613 | CII |
| GSM1942614 | CII |
| GSM1942616 | CII |
| GSM1942617 | CII |
| GSM1942618 | CII |
| GSM1942620 | CII |
| GSM1942624 | CII |
| GSM1942632 | CII |
| GSM1942633 | CII |

Table S19. Results of DEAG clusters in Sarcoidosis

| ID | DEAG cluster |
| --- | --- |
| GSM2203587 | CI |
| GSM2203588 | CI |
| GSM2203589 | CI |
| GSM2203590 | CI |
| GSM2203591 | CI |
| GSM2203592 | CI |
| GSM2203593 | CI |
| GSM2203594 | CI |
| GSM2203595 | CI |
| GSM2203596 | CI |
| GSM2203597 | CI |
| GSM2203600 | CI |
| GSM2203601 | CI |
| GSM2203602 | CI |
| GSM2203603 | CI |
| GSM2203605 | CI |
| GSM2203606 | CI |
| GSM2203607 | CI |
| GSM2203608 | CI |
| GSM2203609 | CI |
| GSM2203610 | CI |
| GSM2203611 | CI |
| GSM2203612 | CI |
| GSM2203613 | CI |
| GSM2203614 | CI |
| GSM2203615 | CI |
| GSM2203616 | CI |
| GSM2203618 | CI |
| GSM2203619 | CI |
| GSM2203620 | CI |
| GSM2203621 | CI |
| GSM2203622 | CI |
| GSM2203623 | CI |
| GSM2203624 | CI |
| GSM2203625 | CI |
| GSM2203626 | CI |
| GSM2203627 | CI |
| GSM2203628 | CI |
| GSM2203629 | CI |
| GSM2203630 | CI |
| GSM2203631 | CI |
| GSM2203632 | CI |
| GSM2203633 | CI |
| GSM2203634 | CI |
| GSM2203635 | CI |
| GSM2203636 | CI |
| GSM2203637 | CI |
| GSM2203640 | CI |
| GSM2203641 | CI |
| GSM2203642 | CI |
| GSM2203644 | CI |
| GSM2203645 | CI |
| GSM2203646 | CI |
| GSM2203647 | CI |
| GSM2203695 | CI |
| GSM2203698 | CI |
| GSM2203701 | CI |
| GSM2203702 | CI |
| GSM2203705 | CI |
| GSM2203712 | CI |
| GSM2203716 | CI |
| GSM2203736 | CI |
| GSM2203739 | CI |
| GSM2203741 | CI |
| GSM479920 | CI |
| GSM479924 | CI |
| GSM479934 | CI |
| GSM479935 | CI |
| GSM479944 | CI |
| GSM479949 | CI |
| GSM479951 | CI |
| GSM479952 | CI |
| GSM479957 | CI |
| GSM479959 | CI |
| GSM479962 | CI |
| GSM479963 | CI |
| GSM479965 | CI |
| GSM479968 | CI |
| GSM479969 | CI |
| GSM479971 | CI |
| GSM479974 | CI |
| GSM479977 | CI |
| GSM479981 | CI |
| GSM2203598 | CII |
| GSM2203599 | CII |
| GSM2203604 | CII |
| GSM2203617 | CII |
| GSM2203638 | CII |
| GSM2203639 | CII |
| GSM2203643 | CII |
| GSM2203693 | CII |
| GSM2203694 | CII |
| GSM2203696 | CII |
| GSM2203697 | CII |
| GSM2203699 | CII |
| GSM2203700 | CII |
| GSM2203703 | CII |
| GSM2203704 | CII |
| GSM2203706 | CII |
| GSM2203707 | CII |
| GSM2203708 | CII |
| GSM2203709 | CII |
| GSM2203710 | CII |
| GSM2203711 | CII |
| GSM2203713 | CII |
| GSM2203714 | CII |
| GSM2203715 | CII |
| GSM2203717 | CII |
| GSM2203718 | CII |
| GSM2203719 | CII |
| GSM2203720 | CII |
| GSM2203721 | CII |
| GSM2203722 | CII |
| GSM2203723 | CII |
| GSM2203724 | CII |
| GSM2203725 | CII |
| GSM2203726 | CII |
| GSM2203727 | CII |
| GSM2203728 | CII |
| GSM2203729 | CII |
| GSM2203730 | CII |
| GSM2203731 | CII |
| GSM2203732 | CII |
| GSM2203733 | CII |
| GSM2203734 | CII |
| GSM2203735 | CII |
| GSM2203737 | CII |
| GSM2203738 | CII |
| GSM2203740 | CII |
| GSM479917 | CII |
| GSM479919 | CII |
| GSM479921 | CII |
| GSM479922 | CII |
| GSM479923 | CII |
| GSM479925 | CII |
| GSM479926 | CII |
| GSM479927 | CII |
| GSM479928 | CII |
| GSM479931 | CII |
| GSM479932 | CII |
| GSM479933 | CII |
| GSM479936 | CII |
| GSM479937 | CII |
| GSM479939 | CII |
| GSM479940 | CII |
| GSM479941 | CII |
| GSM479942 | CII |
| GSM479943 | CII |
| GSM479945 | CII |
| GSM479946 | CII |
| GSM479947 | CII |
| GSM479948 | CII |
| GSM479953 | CII |
| GSM479954 | CII |
| GSM479956 | CII |
| GSM479958 | CII |
| GSM479960 | CII |
| GSM479961 | CII |
| GSM479964 | CII |
| GSM479966 | CII |
| GSM479967 | CII |
| GSM479970 | CII |
| GSM479972 | CII |
| GSM479973 | CII |
| GSM479975 | CII |
| GSM479976 | CII |
| GSM479979 | CII |
| GSM479980 | CII |
